# Biomimetic Thermal Safety Strategies in Batteries for Electric Vehicles: from Biological Principles to Engineering Approaches

**DOI:** 10.1002/advs.202505882

**Published:** 2025-09-28

**Authors:** Weifeng Li, Zhongchun Wang, Yao Xue, Zhenhai Gao, Huanli Sun, Ximin Zhai, Deping Wang, Yupeng Chen

**Affiliations:** ^1^ National Key Laboratory of Automotive Chassis Integration and Bionics Jilin University Changchun 130025 China; ^2^ College of Automotive Engineering Jilin University Changchun 130025 China; ^3^ General Research and Development Institute China FAW Corporation Limited Changchun 130013 China; ^4^ College of Materials Science and Technology Beijing Forestry University Beijing 100083 China

**Keywords:** batteries, bionic design, thermal runaway, thermal safety strategies

## Abstract

With the rapid development of renewable energy and the widespread adoption of electric vehicles, thermal runaway (TR) in batteries has become a critical safety concern. Despite various protective technologies, TR remains frequent due to challenges in metal dendrite growth, material stability, and efficient thermal management. Inspired by natural structures and functions, biological principles have been abstracted to guide novel biomimetic approaches. This review focuses on TR mechanisms, summarizes the corresponding biomimetic principles, and discusses their applications in functional design strategies to enhance battery safety and stability. First, the review outlines current TR protection designs from both intrinsic and system safety perspectives, analyzing biomimetic strategies to enhance intrinsic safety by improving the thermal stability of battery components to reduce the risk of TR. Next, it explores the application of biomimetic designs in thermal management and protection mechanisms, including innovations in thermal management and structure. Finally, this review consolidates the findings from the preceding sections on biomimetic designs for TR protection, emphasizing current challenges and potential future directions, to offer new technical insights and guidance for research on thermal safety in batteries.

## Graphical abstract

Biological principles derived from natural structures and functions inspire novel biomimetic strategies for battery thermal safety. This review summarizes applications of biomimetic concepts in preventing thermal runaway, highlighting design strategies that enhance thermal safety protection. It further aims to stimulate innovative thinking by drawing lessons from nature, providing guidance for the future development of safer battery systems.

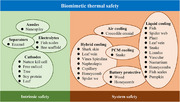

## 1 Introduction

In the context of global efforts to combat climate change and achieve net‐zero emissions, electric vehicles (EVs) have emerged as a pivotal technology for reducing carbon emissions in the transportation sector.^[^ ^1^, ^2^, ^3^ ^]^ The performance and safety of power batteries, the core energy source of EVs, have garnered significant attention.^[^ ^4^ ^]^ To meet the increasing demands for extended driving range and enhanced performance, the specific energy of power batteries continues to rise.^[^ ^5^ ^]^ However, this advancement also elevates the risk of thermal runaway (TR), a hazardous condition that can lead to battery fires or even explosions, posing severe threats to vehicle safety and occupant well‐being. The challenge of TR has become a major obstacle to the large‐scale adoption of EVs.^[^ ^6^ ^]^ Consequently, the thermal safety of power batteries has become a focal point of research and development within both academia and industry worldwide, driving the need for innovative approaches to mitigate these risks and ensure the safe deployment of next‐generation battery technologies.

**Figure 1:**
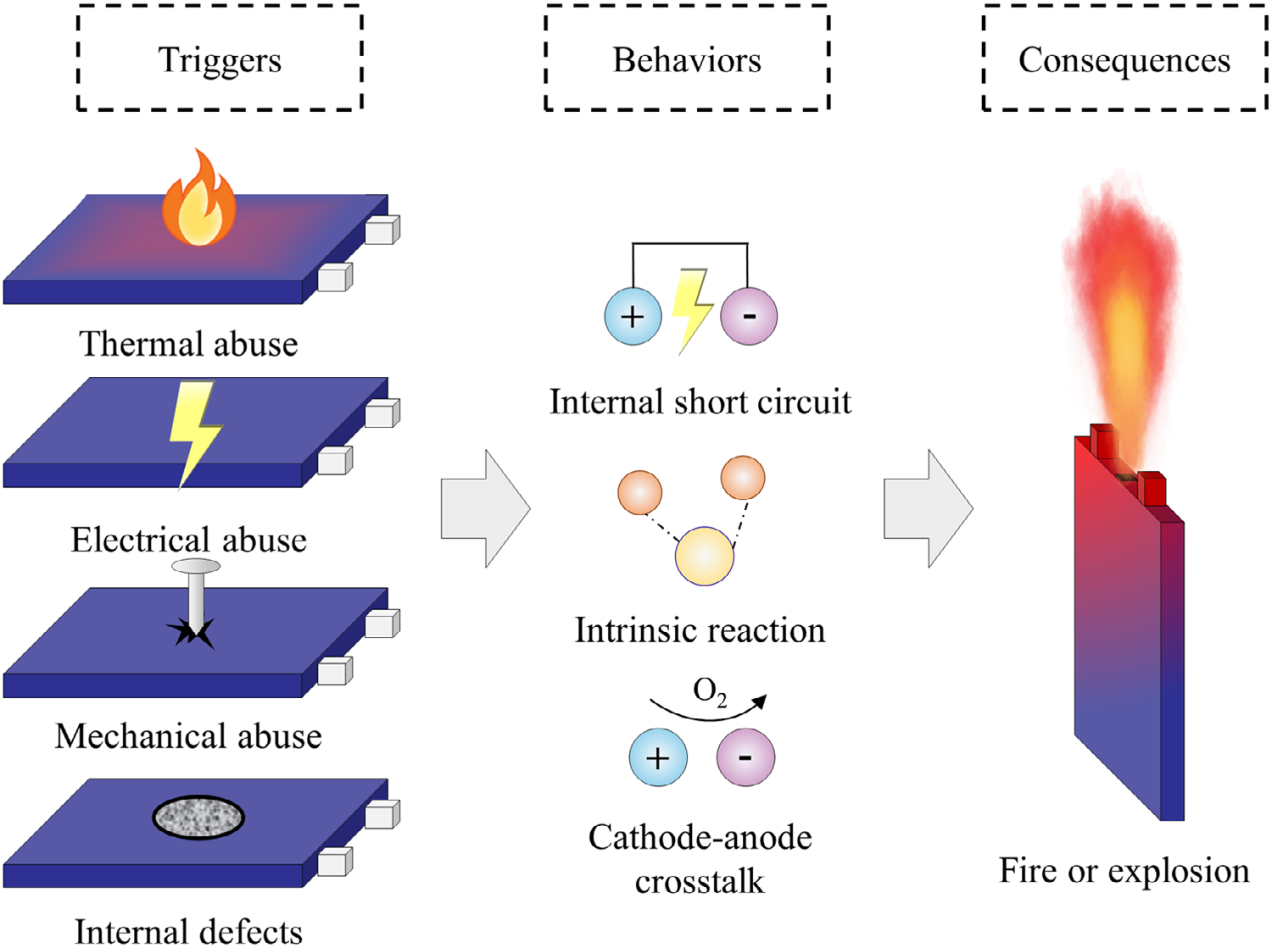
Schematic illustration of the simplified thermal runaway process in batteries.

TR is widely recognized as the most severe safety hazard in power batteries, essentially resulting from the accumulation of heat inside the cell and the initiation of an uncontrollable positive feedback reaction chain.^[^ ^7^ ^]^ In general, TR is triggered by either external abuse or internal defects; once the heat generation rate significantly exceeds the dissipation rate, the local temperature rises sharply, continuously activating side reactions that release additional heat, thereby causing an exponential temperature escalation.^[^ ^8^ ^]^ Typical initiating factors include electrical abuse, thermal abuse, and mechanical abuse.^[^ ^9^, ^10^, ^11^ ^]^ In addition, manufacturing defects may also become potential triggers during long‐term service (**Figure** **1**).^[^ ^12^ ^]^

Under electrical abuse conditions, such as overcharging, the anode potential increases, leading to Cu dissolution and subsequent dendrite deposition on the cathode. These dendrites eventually penetrate the separator, inducing internal short circuits and releasing large amounts of Joule heat.^[^ ^13^ ^]^ In thermal abuse scenarios, when the temperature reaches ≈80 °C, the solid electrolyte interphase (SEI) begins to decompose, releasing gases. Further temperature elevation triggers electrolyte decomposition and oxygen release from cathode materials, while high‐temperature melting of the separator causes large‐scale internal short circuits, thereby forming a typical thermal reaction chain.^[^ ^14^ ^]^ This process is dominated by chemical reactions and thermal degradation, in contrast to the more physically induced mechanisms of other abuse conditions. In the case of mechanical abuse, external crushing or penetration directly compromises the separator's physical barrier, leading to rapid internal short circuits and abrupt temperature rise.^[^ ^15^ ^]^

Throughout this evolution, gas generation exhibits distinct staged characteristics. Initially, the decomposition of the SEI and electrolyte produces small molecular gases such as CO_2_, CO, and CH_4_. As the temperature further increases, hydrocarbon gases, H_2_, and oxygen‐enriched atmospheres gradually accumulate. At elevated temperatures, oxygen release from the cathode and the thermal cracking of organic components jointly drive violent gas emission. These gases are not only the direct cause of the rapid rise in internal cell pressure but also critical factors determining the intensity and flammability of jetting. During venting, electrolyte aerosols and carbonized particulates are frequently entrained, which, when expelled at high velocity, readily form flame plumes and secondary combustion.^[^ ^16^, ^17^, ^18^ ^]^

It is evident that regardless of the abuse scenario, the outcome may involve large‐scale internal short circuits and intense exothermic reactions, accompanied by the generation of substantial amounts of high‐temperature combustible gases. Within sealed cells, these gases accumulate rapidly, raising internal pressure and, during venting, entraining electrolyte droplets and carbonized particles to form highly combustible jets.^[^ ^19^, ^20^, ^21^ ^]^ The ejected gases and particulates from a single cell can directly ignite the cell itself or nearby materials, and may also spread within modules/packs to neighboring cells, creating new ignition sources that drive thermal propagation.^[^ ^22^, ^23^ ^]^ Therefore, the generation, composition, accumulation, and jetting of gases are not only key manifestations of TR but also critical bridges linking single‐cell failure to system‐level disasters. Nevertheless, current research on the regulation of TR gases remains relatively limited. Nevertheless, although various engineering strategies have been developed to suppress TR gas generation, bio‐inspired designs specifically targeting gas regulation remain absent, representing a promising direction for future research.^[^ ^16^ ^]^

**Figure 2:**
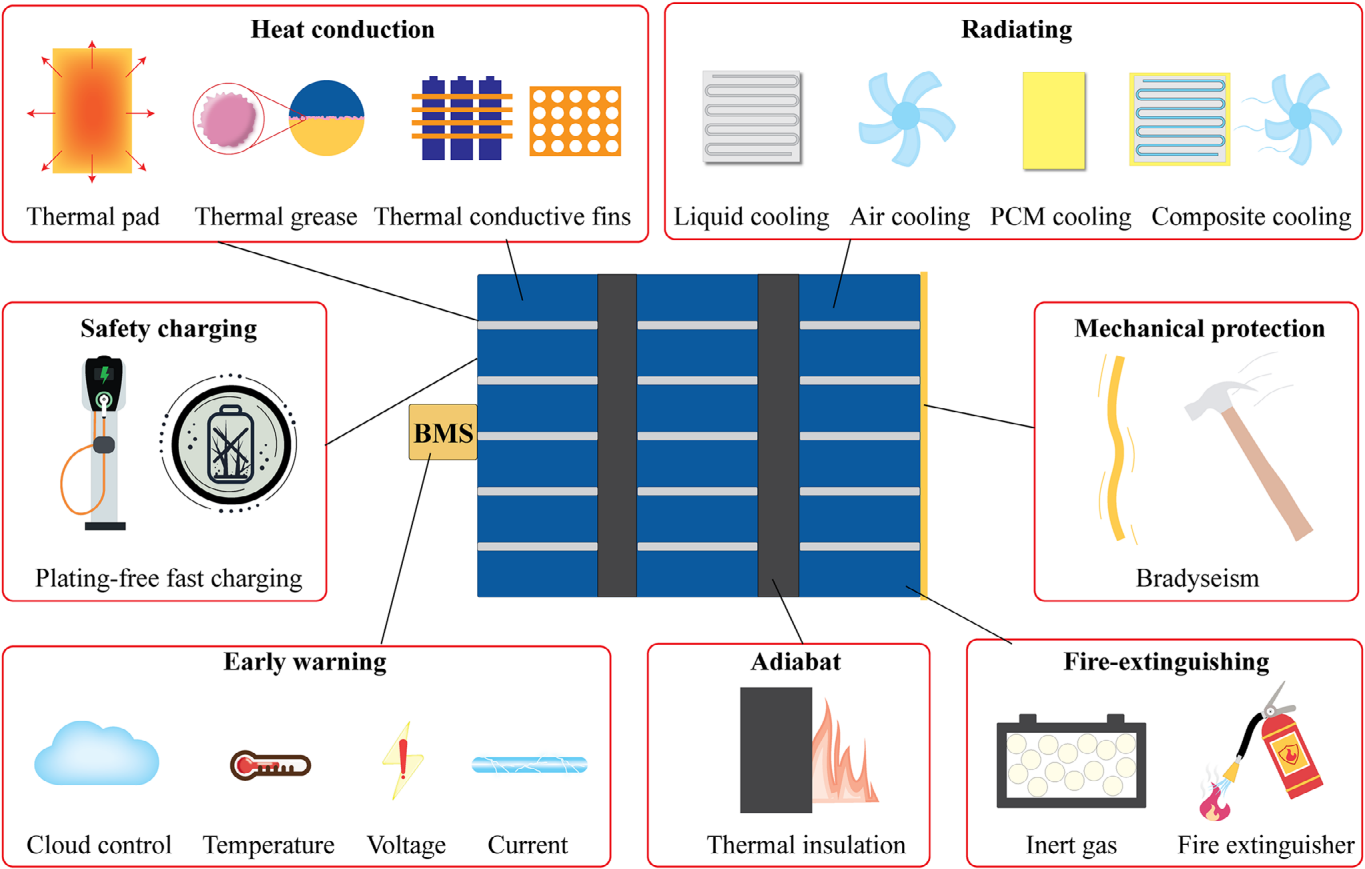
System safety approaches for TR prevention in batteries.

Existing TR protection strategies can be broadly categorized into two types: system safety and intrinsic safety.^[^ ^24^, ^25^ ^]^ As shown in **Figure** **2**, system safety primarily involves external design and management methods to provide early warnings of TR, suppress its occurrence, and prevent heat propagation.^[^ ^26^ ^]^ This approach focuses on ensuring the overall safety of the battery through monitoring, control, and optimization of system design. For instance, a battery management system (BMS) continuously monitors parameters such as voltage, temperature, and current to ensure the battery operates within a safe range.^[^ ^27^ ^]^ When anomalies are detected, the BMS takes timely action. Concurrently, a thermal management system regulates the operating temperature of the battery to prevent localized overheating that could lead to safety issues.^[^ ^28^ ^]^ Thermal management strategies can be divided into three aspects: insulation, thermal conduction, and heat dissipation.^[^ ^24^, ^29^, ^30^ ^]^ Insulation involves introducing insulating materials between battery cells to limit the diffusion of heat after TR, effectively delaying heat conduction and preventing chain reactions in the battery pack. Thermal conduction employs interface materials to rapidly transfer heat to safe zones. Heat dissipation techniques utilize various methods such as air cooling, liquid cooling, phase change material (PCM) cooling, and composite cooling to dissipate heat.^[^ ^31^, ^32^, ^33^, ^34^ ^]^ Furthermore, other system safety designs include lithium‐free fast charging technology, mechanical protection, and onboard fire suppression systems, collectively forming a multi‐layered protection system.^[^ ^35^, ^36^, ^37^ ^]^

**Figure 3:**
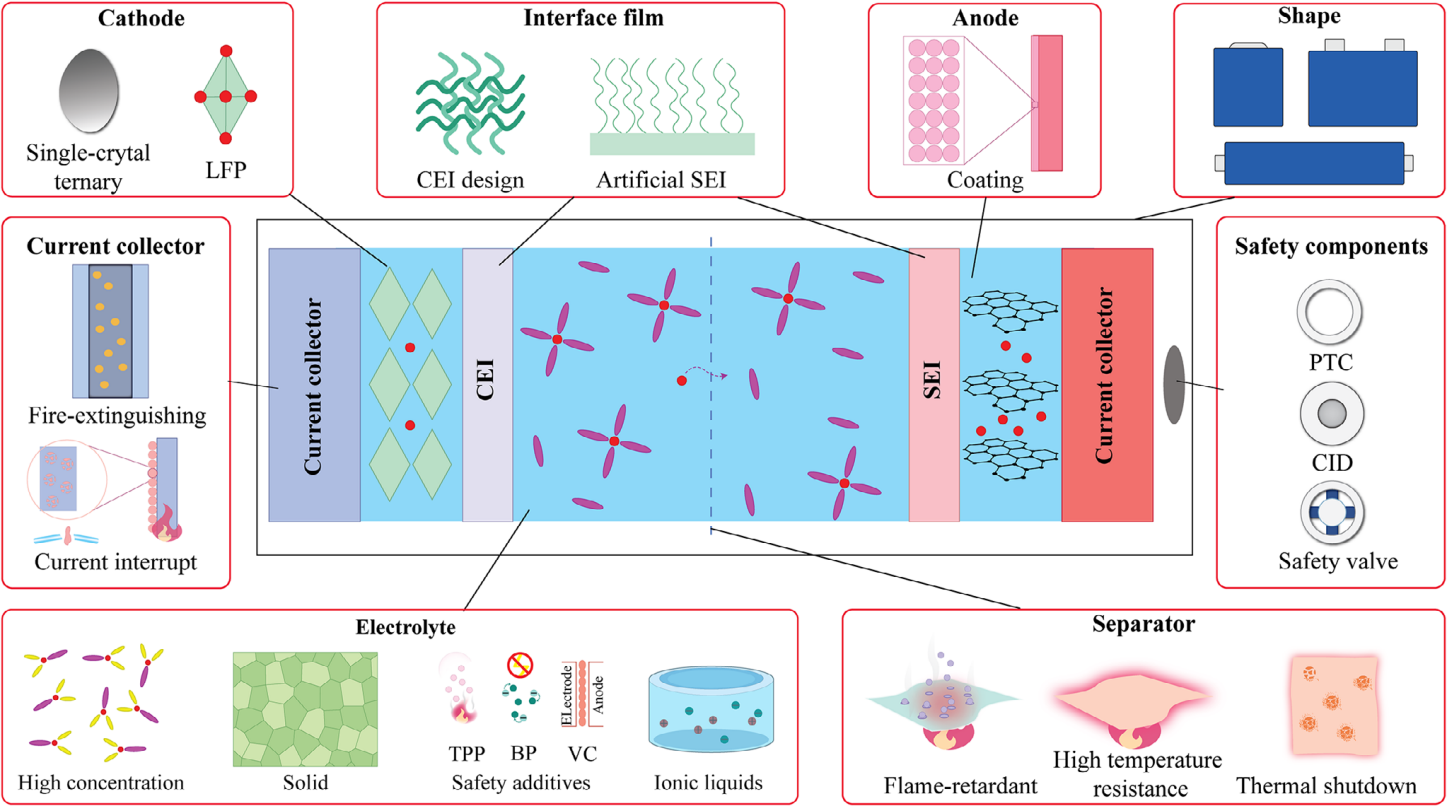
Intrinsic safety approaches for TR prevention in batteries.

In contrast, intrinsic safety places greater emphasis on optimizing the internal materials and structures of the battery, aiming to reduce the likelihood of TR from the source.^[^ ^38^ ^]^ As shown in **Figure** **3**, intrinsic safety strategies directly target the battery's inherent design, focusing on the stability of materials and chemical reactions to ensure that the battery remains resistant to TR even under extreme conditions.^[^ ^39^ ^]^ For instance, when selecting cathode materials, options like single‐crystal ternary materials or lithium iron phosphate can be employed. In terms of internal interface design, artificially synthesized, high‐safety cathode electrolyte interface and solid electrolyte interface films can be utilized.^[^ ^30^, ^40^, ^41^, ^42^ ^]^ Furthermore, coating techniques can be applied to anode materials to enhance safety.^[^ ^43^ ^]^

Additionally, the design of the battery casing is a crucial aspect of battery safety, with internal safety components such as positive temperature coefficient (PTC) devices, current interrupt devices (CID), and safety valves playing an effective role in risk prevention.^[^ ^44^, ^45^, ^46^ ^]^ In the design of separators, flame retardants can be added, or high‐temperature resistant separators can be employed, and separators with thermal barrier functions can even be introduced.^[^ ^47^, ^48^, ^49^, ^50^ ^]^ At the electrolyte level, the utilization of high‐concentration electrolytes, solid electrolytes, ionic liquids, and safe additives can significantly enhance thermal stability.^[^ ^5^, ^6^, ^51^, ^52^, ^53^ ^]^ Regarding current collectors, the incorporation of fire‐extinguishing agents or the coating of microspheres can effectively release extinguishing agents when temperatures reach a threshold, thereby suppressing the fire source, while microspheres dissolve to interrupt the current flow.^[^ ^54^ ^]^

Despite the significant advancements achieved through the aforementioned TR protection technologies, electric vehicle fires triggered by TR in power batteries continue to occur, indicating that fire suppression effectiveness is still lacking. The thermal safety of power batteries has become a critical bottleneck in the development of new energy vehicles. Therefore, there is an urgent need to develop more effective prevention and control strategies to achieve revolutionary progress in thermal safety design, ensuring the safe operation of batteries and promoting the sustainable development of the electric vehicle industry.

Through long‐term evolution, organisms in nature have developed a series of complex structures and mechanisms to cope with extreme environments, providing invaluable inspiration for modern engineering design.^[^ ^55^ ^]^ The core principle of biomimicry lies in drawing wisdom from nature and applying biological principles to industrial design, thereby driving technological advancements across multiple fields, particularly in enhancing material strength, durability, and efficiency.^[^ ^56^, ^57^ ^]^ Significant breakthroughs have been achieved in various industrial sectors through biomimetic approaches, such as the application of bionic sensors in environmental monitoring, the flexible operation of soft robots in complex environments, and the tactile feedback technology of bionic skin in medical.^[^ ^58^, ^59^, ^60^ ^]^ These biomimetic strategies have substantially improved the performance and lifespan of industrial products.^[^ ^61^ ^]^ With the growing global energy demand and the advocacy for a low‐carbon economy, biomimicry also shows promising applications in energy storage.

As a method inspired by the self‐protection and adaptive mechanisms of organisms in nature, biomimetic design has garnered widespread attention in the field of power battery safety in recent years.^[^ ^55^, ^62^ ^]^ Biomimetic radiator inspired by crocodile structure, which offers rich inspiration for the thermal safety design of batteries.^[^ ^63^ ^]^ By simulating these characteristics, researchers have proposed various biomimetic strategies aimed at enhancing both the intrinsic and system safety of power batteries.^[^ ^64^, ^65^ ^]^ Although some of these technologies were introduced years ago, further research continues to reveal the potential impacts of natural biological mechanisms. Therefore, this review not only aims to summarize the applications of biomimetic concepts in battery thermal safety protection in recent years but also seeks to inspire innovative thinking through lessons drawn from nature, guiding the development of battery thermal safety design.

**Figure 4:**
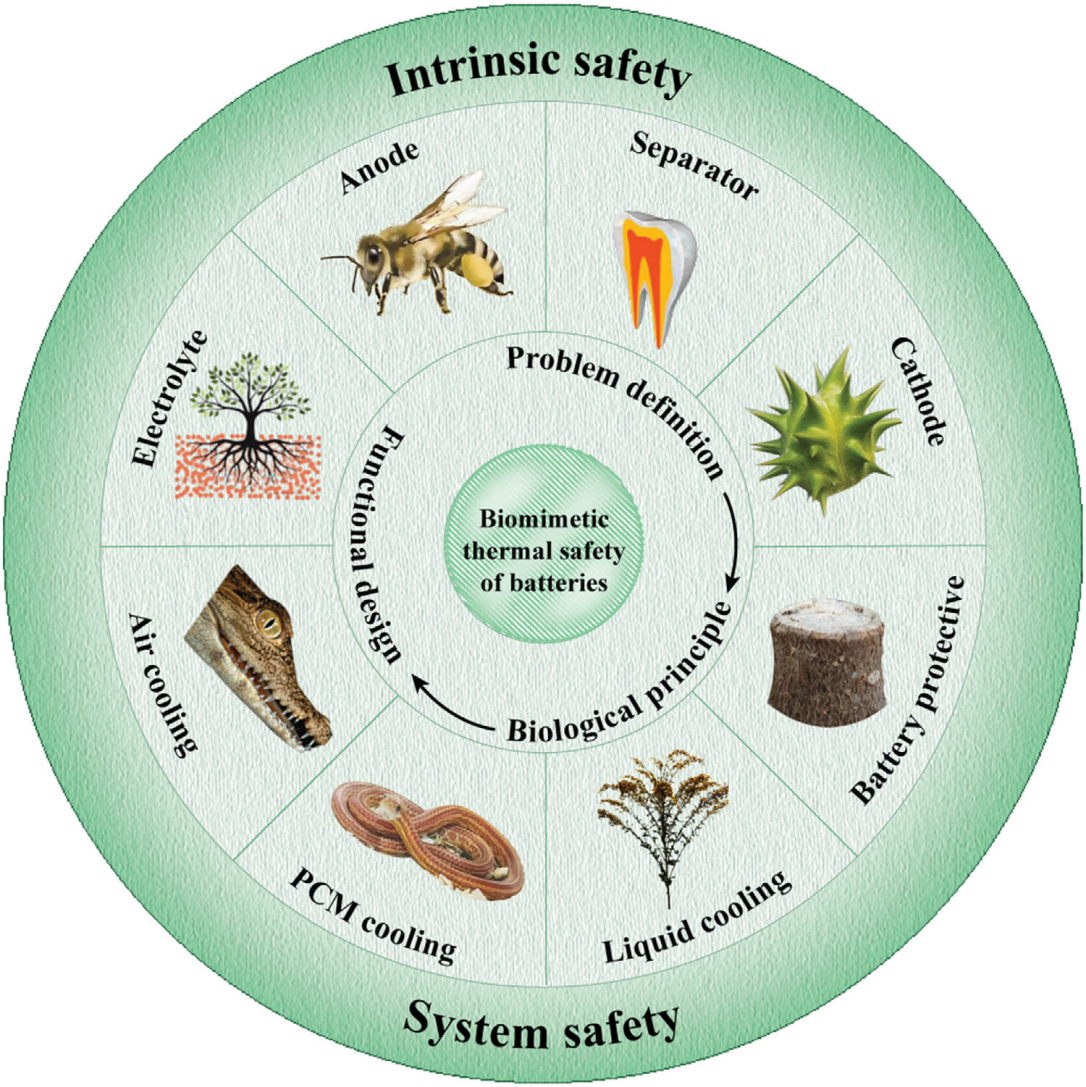
Typical bionic strategies for intrinsic safety and system safety of batteries.The image of the tooth is adapted with permission.^[^ ^85^ ^]^ Copyright 2022, Elsevier B.V. The image of the tree is adapted with permission. ^[^ ^80^ ^]^ Copyright 2023, Wiley‐VCH GmbH. The image of the crocodile is adapted with permission.^[^ ^117^ ^]^ Copyright 2025, the authors. The image of the honeybee is adapted with permission.^[^ ^120^ ^]^ Copyright 2022, Elsevier Inc. The image of the datura stramonium seed is adapted with permission.^[^ ^119^ ^]^ Copyright 2025, the authors.

The focus will first be on the application of biomimetic design in the intrinsic safety of power batteries, with an emphasis on how material selection affects battery thermal safety performance, as shown in **Figure** **4**. The next section will discuss the role of biomimetic design in system safety, elaborating on how biomimetic strategies can optimize the thermal management and protection mechanisms of battery systems. Finally, the future application prospects of biomimetic design in the thermal safety of power batteries will be explored, along with an analysis of unresolved challenges and issues in current research. It is envisioned that this review will provide valuable references and technical insights for the research and practice of power battery safety design.

## 2 Intrinsic Safety Biomimetic Design for Batteries

Intrinsic safety‐design of batteries refers to the design of materials or battery components from within the battery itself. In recent years, numerous research teams have engaged in in‐depth exploration and practice in the field of biomimetic design for intrinsic battery safety, yielding a series of positive results.^[^ ^55^ ^]^ For instance, by mimicking cellular structures and drawing inspiration from the mechanisms by which natural killer (NK) cells recognize foreign entities, the methods that biological organisms use to eliminate free radicals, layered structures found in nature, the robust architecture of enamel, the microscopic morphology of bee hairs and leg hairs, as well as the hydrophobic properties of fish scales, researchers have developed theoretical guidelines and foundations for the design of materials and components within batteries, leading to significant achievements.^[^ ^66^, ^67^ ^]^

### 2.1 Cathodes

The cathode is often the initial trigger of TR, as high‐voltage operation can induce oxygen release and accelerate exothermic reactions with the electrolyte.^[^ ^68^ ^]^ Once such reactions occur, internal heat generation rises sharply and may cascade into uncontrollable failure.^[^ ^69^ ^]^ To mitigate these risks, thermal response materials such as PTC composites have been investigated. PTC materials can increase resistance with temperature and suppress heat generation, but their conventional forms usually suffer from slow switching, high filler content, and limited conductivity, which compromise energy density and mechanical flexibility.^[^ ^70^ ^]^ These limitations underscore the need for advanced materials that can respond rapidly and reliably to thermal events.

#### 2.1.1 Inspired by Natural Hierarchical Structures: Nanospiked Nickel‐Based Thermal Responsive Polymer Switch Materials

**Figure 5:**
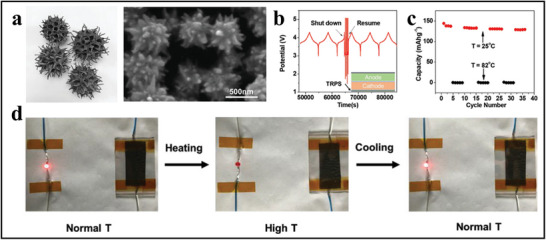
Bionic illustration and thermal performance of nanospiked nickel‐based TRPS. (a) SEM micrographs of uniformly structured nanospiky nickel (right), inspired by the spiky morphology of jimson weed (left).  Adapted with permission.^[^ ^71^ ^]^ Copyright 2020, WILEY‐VCH Verlag GmbH & Co. KGaA, Weinheim. (b) Charge‐discharge profiles of an enhanced‐safety NCM111 battery, comparing performance before and after thermal shutdown at 82 °C. Reproduced with permission.^[^ ^71^ ^]^ Copyright 2020, WILEY‐VCH Verlag GmbH & Co. KGaA, Weinheim. (c) Cycle performance of a safety‐enhanced NCM111 battery. Reproduced with permission.^[^ ^71^ ^]^ Copyright 2020, Copyright 2020, WILEY‐VCH Verlag GmbH & Co. KGaA, Weinheim. (d) Thermal switching characteristics of Ni|PVDF composite films demonstrated via hot plate testing. Reproduced with permission.^[^ ^71^ ^]^ Copyright 2020, WILEY‐VCH Verlag GmbH & Co. KGaA, Weinheim.

As shown in **Figure** **5**(a), Li et al. drew inspiration from the layered structures found in nature (such as sweetgum fruit and jimson weeds) to develop thermal‐responsive polymer switch materials (TRPS) based on nano‐spiky nickel particles.^[^ ^71^ ^]^ The nanoscale nickel particles, ≈500 nm in size, are synthesized through aqueous phase reactions and embedded in a polyvinylidene fluoride (PVDF) and polyethylene (PE) matrix to form a highly conductive composite film. Compared to conventional micron‐sized nickel particles, nano‐spiky particles can enhance conductivity even at a volume fraction of less than 5%. At a filler content of 31.8%, the TRPS film achieves a room temperature conductivity of 300 S cm^−1^ and a PTC strength of up to 8, significantly surpassing traditional materials. Furthermore, it can complete resistance switching in under 1 s, with adjustable switching temperatures ranging from 75 to 170 °C. The small size of the nickel particles allows the film thickness to be reduced to 5 µm or even thinner. These characteristics enable the TRPS film to achieve more effective thermal management without compromising battery performance, thus ensuring the safety of high energy density batteries.

In Figure 5(b), battery safety tests are conducted by fabricating lithium‐ion batteries (LIB) with oxide‐based cathodes (LiMn_1/3_Co_1/3_Mn_1/3_O_2_ or NCM111) and TRPS‐based films on the current collectors (as illustrated in the inset of Figure 5(b)). Initially, the LIB cycles at room temperature under normal conditions, showing typical charge/discharge behavior and providing a discharge capacity of 140 mAh·g^−1^ within a voltage range of 3–4.3 V, which is close to the theoretical reversible capacity of NCM111. Once the chamber temperature rises to 82 °C, the battery immediately shuts down due to a sharp increase in resistance, as indicated by a significant and rapid change in battery voltage. When the ambient temperature exceeds 82 °C, the capacity drops to zero, demonstrating that the battery is completely shut off by the TRPS film. Subsequently, once cooled back to room temperature, the battery cell can resume normal cycling, with the battery capacity remaining unchanged. This thermal shutdown can be repeated multiple times without significant change in sensitivity (Figure 5(c)). The stable cycling of the TRPS‐regulated LIB indicates that the Ni|PE film can effectively repeat thermal shutdown without noticeable performance degradation. These characteristics suggest that the Ni|PE‐based TRPS can be utilized for thermal regulation in LIBs to provide effective safety protection.

Figure 5(d) showcases a simple circuit for circuit regulation using the TRPS film's rapid reversible thermal switching, connecting a light‐emitting diode (LED) with a Ni|PVDF‐based TRPS strip. When heated on a hot plate to a specific temperature, the LED can be immediately turned off. Upon cooling, the LED lights up again, indicating that the resistance of the TRPS film undergoes reversible changes with temperature. The switching can be repeated multiple times without reducing sensitivity.

The researchers synthesized nano‐spiky nickel particles as conductive fillers, inspired by the uniformly layered structures found in nature. This biomimetic design provides unique performance advantages. The resulting TRPS composite films exhibit high room temperature conductivity, rapid resistance switching, high PTC strength, adjustable switching temperatures, and good reversibility, with film thickness as thin as 5 µm or less, making them standout materials in their category and offering more effective solutions for battery thermal management.

### 2.2 Electrolytes

The battery electrolyte, crucial for ion transport, directly affects energy density, cycle life, and safety. Commercial lithium‐ion electrolytes, typically composed of carbonate solvents, lithium salts, and additives, suffer from high flammability and poor thermal stability, making them prone to TR under high temperature, overcharging, or internal short circuits. TR risks arise from electrolyte volatilization, thermal decomposition, lithium‐salt instability, low conductivity, and dendrite growth, all of which can trigger severe side reactions or short circuits. Optimizing electrolyte composition and properties is therefore essential to mitigate these hazards.^[^ ^72^, ^73^ ^]^

#### 2.2.1 Liquid Electrolytes

Liquid electrolytes face numerous challenges in the TR protection of batteries. First, they are highly flammable, typically composed of organic solvents and lithium salts. Most organic solvents, such as common carbonate solvents, have low flash points and are prone to combustion, which can trigger and propagate TR. Second, they exhibit poor chemical stability and can easily undergo side reactions with electrode materials under certain conditions.^[^ ^74^ ^]^ For instance, at high voltages, they may undergo oxidative decomposition, producing gases and heat, and they can also react with lithium metal anodes to form unstable SEI films, which can lead to lithium dendrite growth and increase the risk of short circuits.^[^ ^16^, ^72^ ^]^ Furthermore, their thermal stability is inadequate; as the battery temperature rises, the stability of liquid electrolytes significantly decreases, causing organic solvents to decompose and volatilize, releasing flammable gases that exacerbate the risk of TR.^[^ ^75^ ^]^

To address these issues, various efforts can be made at the electrolyte level. On one hand, there is a need to develop novel non‐flammable liquid electrolytes by designing and optimizing the molecular structure of organic solvents to synthesize new solvents with high flash points, low volatility, and non‐flammability. Additionally, flame retardants can be added to the liquid electrolyte to enhance its safety profile.

##### Thermal Immune Microcapsules Inspired by NK Cells

**Figure 6:**
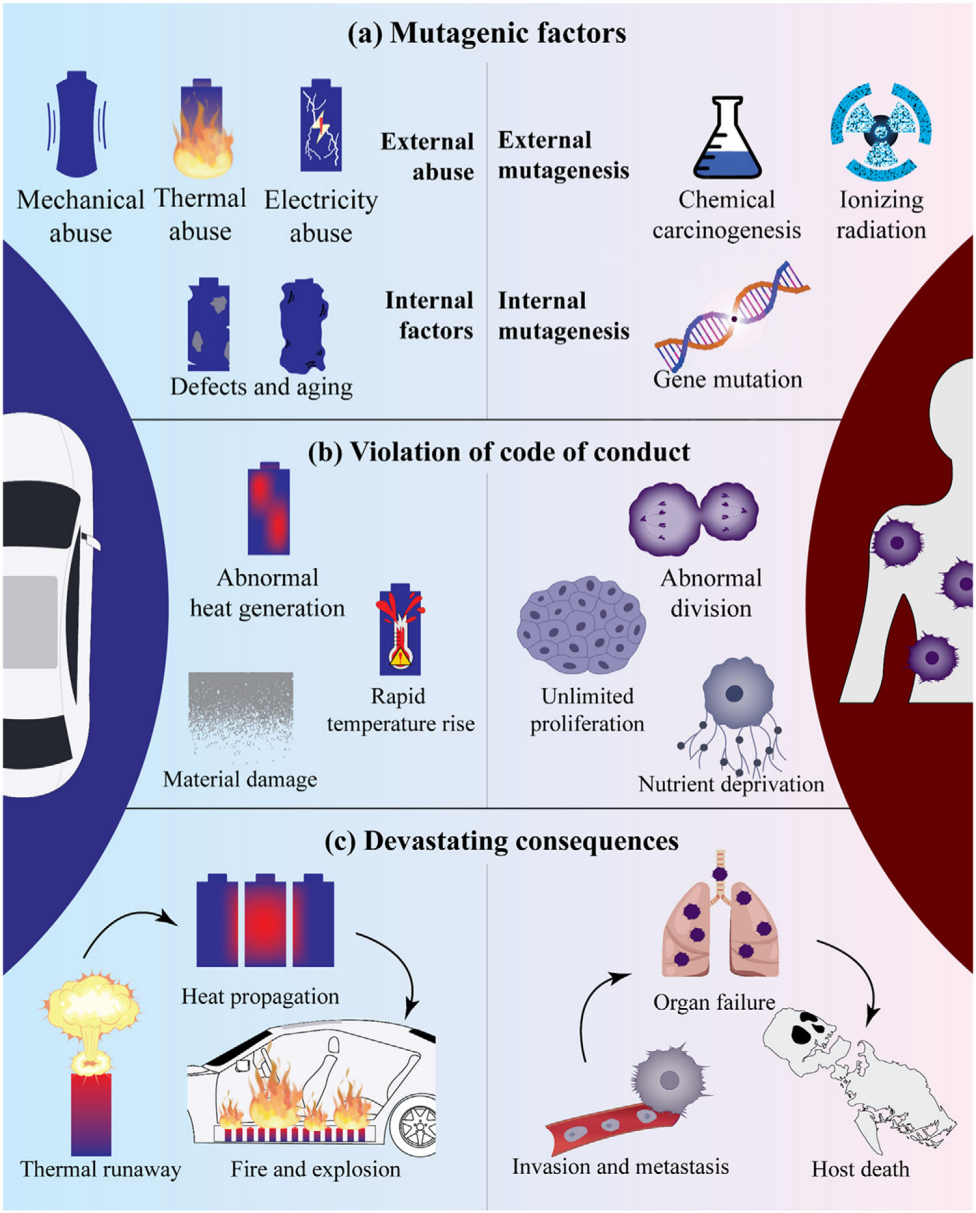
Evolution of similarities between battery thermal runaway and malignant tumor cells in organisms. (a) Internal and external inducing factors. (b) Disruption of code of conduct. (c) Severe consequences.

Battery TR is characterized by abnormal temperature rises that occur at a significantly faster rate than those caused by normal heat generation in batteries. This phenomenon draws parallels to the characteristics of tumor cells in biological organisms. As shown in **Figure** **6**, a further analysis reveals a high degree of similarity between battery TR and tumor cell behavior, specifically in the following aspects:

**(a) Mutagenic factors**. The majority of tumor cells in biological organisms arise from the interaction of environmental and genetic factors. Environmental factors include biological agents (primarily viruses), physical influences (such as various forms of radiation and toxic substances), and chemical agents (carcinogens). These are akin to the triggers of battery TR, which include mechanical abuse, thermal abuse, and electrical abuse. Additionally, inherent genetic mutations within organisms can lead to tumor formation, paralleling how foreign particles contaminating batteries during manufacturing can result in congenital defects that cause internal short circuits, triggering TR.

**(b) Violation of code of conduct**. Under the influence of mutagenic factors, normal cells begin to divide uncontrollably, leading to the formation of tumor cells. Similarly, batteries may undergo abnormal heat generation in the early stages of TR. The fundamental behavior of both systems is characterized by a breakdown in regulatory mechanisms.

***Quantitative Change***. After the initial division, tumor cells begin to proliferate at an accelerated pace, eventually leading to uncontrolled, limitless growth. This mirrors the rapid heat generation resulting from chain reactions during battery TR, where both phenomena transition from slow to rapid increases in quantity.

***Qualitative Change***. As tumors evolve, their vitality increases, enhancing their ability to invade other cells and organs within the organism. Tumors consume nutrients required for the growth of normal cells while metastasizing to damage various organs. In the case of batteries, TR gradually destroys internal structures, leading to a loss of function and normal operation. Both processes culminate in a loss of normal function, resulting in a qualitative change in the biological organism or battery.

**(c) Consequences**. Tumor cells often lead to the death of the organism, while TR in batteries can cause phenomena such as fire and smoke, which may spread throughout the battery system, making it difficult to extinguish the fire and ultimately resulting in battery failure. Both scenarios lead to catastrophic outcomes.

**Figure 7:**
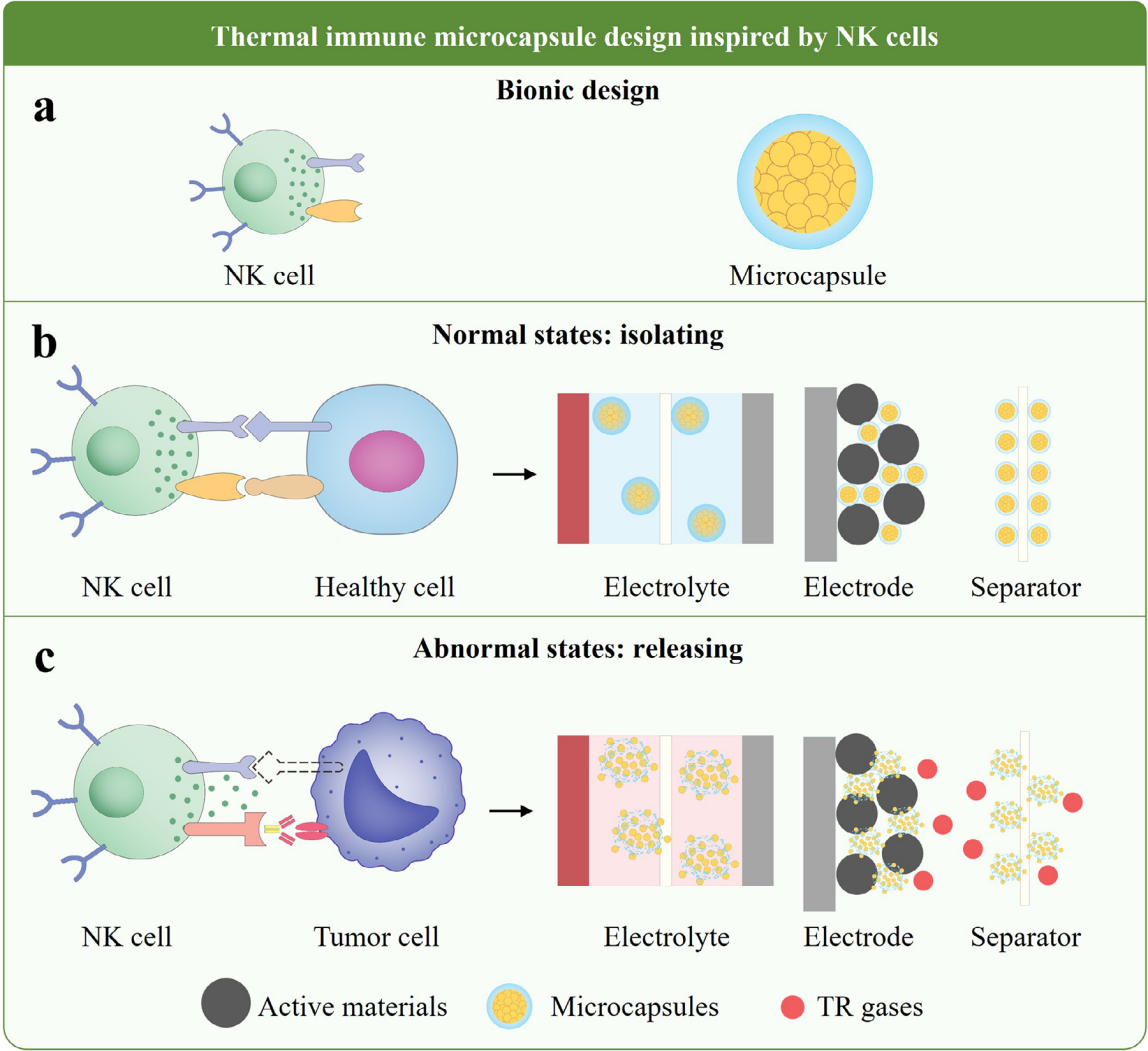
Thermal immune microcapsules design inspired by NK cells. (a) Biomimetic principles. (b) Isolation in normal operation. (c) Release in abnormal conditions.

To combat the uncontrolled proliferation of tumor cells, biological organisms have evolved NK cells, which play a critical role in innate immunity, particularly in early defense mechanisms. NK cells can kill tumor cells without affecting normal cells, paralleling the goal for battery systems: to suppress TR while maintaining normal operation. This poses a significant challenge in battery safety design. NK cells, which are micrometer‐sized and possess a core‐shell structure, can release cytotoxic agents to destroy tumor cells when they are identified while refraining from releasing these agents around normal cells, ensuring that standard functions are not impaired as illustrated in **Figure** **7**. This mechanism of isolation and release, along with the core‐shell structure of NK cells, appears to provide a promising direction for the design of batteries with TR immunity. The concept involves enabling batteries to evolve a mechanism akin to that of NK cells, thereby achieving TR immunity.

Based on the isolation‐release mechanism of NK cells in anti‐tumor immunity, a concept can be developed for designing thermal immune substances (TIS) to address TR in batteries. The design of TIS involves several key aspects:

***Scale***. The TIS should operate at the cellular level, specifically within the micro‐ and nanoscale range.

***Structure***. Similar to NK cells, TIS will have a core‐shell structure. The outer shell will be made from materials that are highly compatible with battery components, while the core will contain substances that suppress TR. This design prevents the TR suppressants from coming into direct contact with the electrolyte, thereby preserving the electrochemical performance of the battery during normal operation.

***Functionality***. In the event of TR in a LIB, the TIS will release the TR suppressants through the outer shell to mitigate or prevent further TR. This mechanism aims to achieve high safety and high performance, effectively addressing the current challenges in battery safety and prevention.

This paper refers to the TR suppressants with a core‐shell structure and isolation‐release mechanism at the micro‐ and nanoscale as “thermal immune micro‐nanocapsules”. As shown in Figure 7, these capsules can be incorporated into the electrolyte, coated onto the electrodes, or applied to the separators, dispersing throughout the battery materials, akin to how NK cells circulate within the bloodstream.

To effectively mitigate TR in batteries, it is essential to identify substances that can act as “killing agents” to suppress the onset of such events. These substances should ideally be cost‐effective and include common flame‐retardant additives, such as triphenyl phosphate (TPP), which can reduce the flammability of electrolytes, potentially rendering them non‐flammable. Additionally, TPP can release compounds that capture combustion free radicals, thus interrupting the burning process. Other suitable candidates may include amine‐based substances that deactivate the electrolyte's activity.^[^ ^76^, ^77^ ^]^

The second, more critical, yet challenging aspect is finding materials that mimic cellular membranes. These materials must not adversely affect the performance of the battery components they contact, a property known as “electrochemical compatibility”, ensuring no negative side effects occur. Furthermore, these materials need to melt or rupture during TR to promptly release encapsulated flame retardants and other “killing agents.”

Finally, it is necessary to consider how to encapsulate these “killing agents” within the previously mentioned shell materials, allowing for their mixing with the electrolyte or coating onto the separator. The resulting core‐shell structured particles must achieve a size on the micrometer or nanometer scale, akin to cellular dimensions, to minimize any significant impact on the internal resistance of the battery. Currently, microencapsulation technology can achieve this objective, enabling the creation of flame‐retardant microcapsules for batteries.

These microcapsules are formed by coating solid or liquid substances with film‐forming materials to create small particles ranging from nanometer to several hundred micrometers in diameter, effectively reaching the cellular scale. This process can be understood as encapsulating one substance within another to form core‐shell structured microspheres at the micro‐ and nanoscale. There are several established methods for preparing these microcapsules, such as polymerization and phase separation techniques.

**Figure 8:**
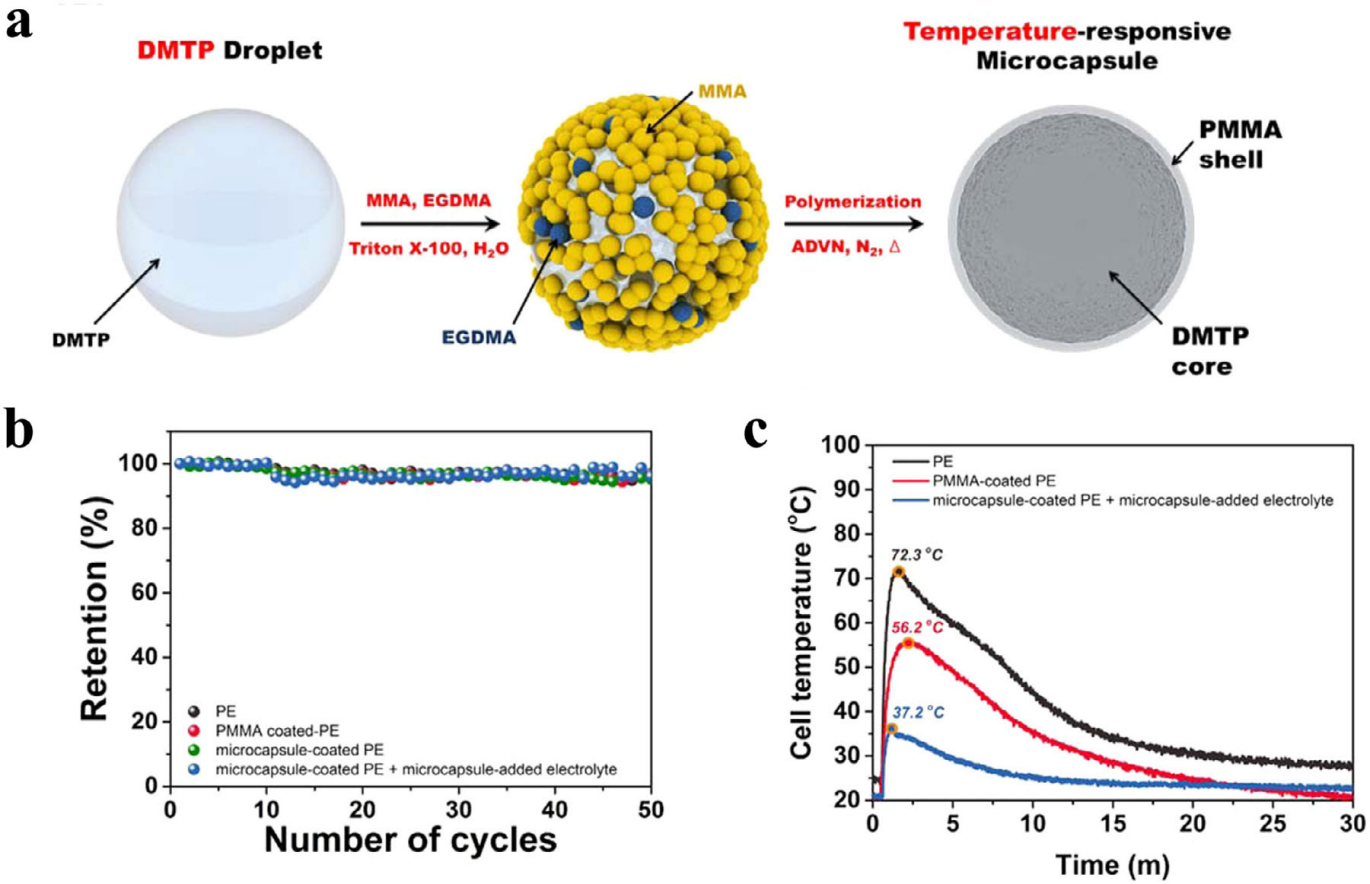
Preparation of DMTP microcapsules and their performance evaluation in batteries. (a) A rigid PMMA shell was formed around DMTP droplets through an oil‐in‐water emulsion polymerization process, utilizing methyl methacrylate as the monomer, ethylene glycol dimethacrylate as the cross‐linker, and 2,2‐azobis(2,4‐dimethylvaleronitrile) as the initiator. Reproduced with permission.^[^ ^78^ ^]^ Copyright 2015, NANO letters. (b) Cycling performance evaluated over 50 cycles to assess capacity retention. Reproduced with permission.^[^ ^78^ ^]^ Copyright 2015, NANO letters. (c) Thermal behavior recorded during nail penetration tests on fully charged graphite|NCM523 cells. Reproduced with permission.^[^ ^78^ ^]^ Copyright 2015, NANO letters.

As proposed by Yim et al., a novel approach to enhance the self‐extinguishing capability of LIBs involves integrating temperature‐responsive microcapsules containing fire extinguishing agents, effectively improving safety.^[^ ^78^ ^]^ As illustrated in **Figure** **8**(a), flame‐retardant microcapsules can be prepared using a water‐in‐oil emulsion polymerization process, where poly(methyl methacrylate) (PMMA) ethylene glycol dimethacrylate serves as the shell and 1,1,1,2,2,3,4,5,5,5‐decafluoro‐3‐methoxy‐4‐(trifluoromethyl)pentane) (DMTP) as the core. These microcapsules are designed to release extinguishing agents when the internal temperature of the LIB rises, facilitating an endothermic reaction that quickly absorbs heat and suppresses further temperature increases, thus preventing undesirable TR.

As shown in Figure 8(b), the addition of flame‐retardant microcapsules does not significantly affect the cycling performance of the battery, with a capacity retention exceeding 95% after 50 cycles. Moreover, the safety of cells equipped with self‐extinguishing microcapsules is markedly enhanced during nail penetration tests, as demonstrated in Figure 8(c).

Despite the significant advancements and promising application prospects of flame‐retardant microcapsule technology related to thermal immune batteries, certain limitations still exist. The encapsulation effect of the microcapsule shell can lead to a delayed release of the flame retardants, thereby impacting the immediate responsiveness during fire incidents or battery TR events. Typically, the flame retardants must reach specific temperature or pressure thresholds to be released, which may not be achievable during the early stages of a fire, hindering their ability to swiftly suppress flames or reduce temperatures. Additionally, the thermal stability and decomposition characteristics of shell materials further influence the precision of the release control. Notably, some high‐temperature‐resistant shell materials may not rupture or dissolve at lower temperatures, delaying the timely release of flame retardants and diminishing their efficacy. For automotive power batteries, this delay could result in ineffective suppression of initial TR, increasing the risk of fire propagation. Therefore, achieving precise control over the release of flame‐retardant microcapsules, ensuring their responsiveness under specific temperature or environmental conditions, poses higher demands on current microcapsule fabrication processes.

**Figure 9:**
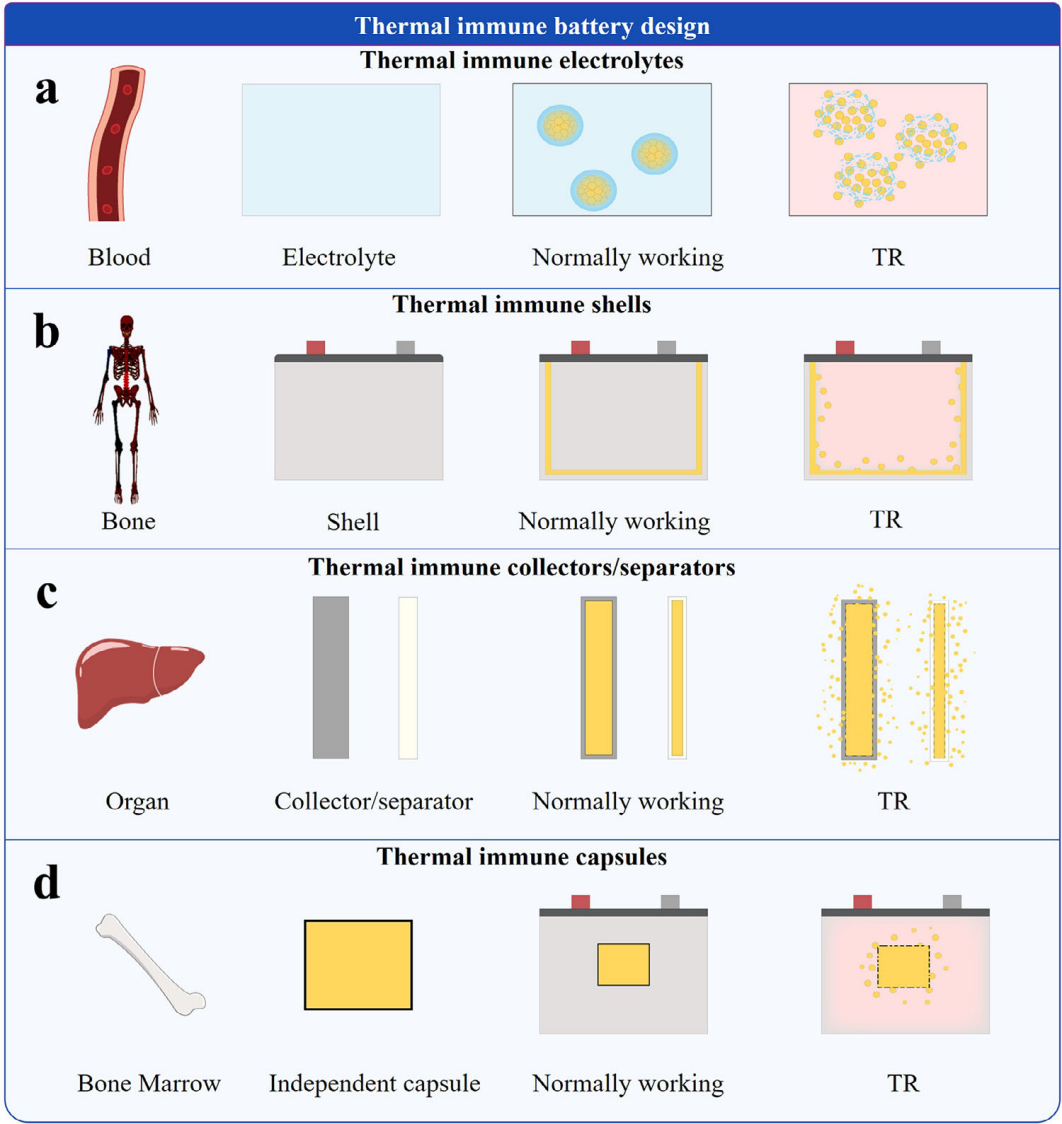
Thermal immune battery design: biomimetic strategies inspired by NK cell localization. (a) Blood‐inspired thermal immune electrolyte design. (b) Bone‐inspired thermal immune shell design. (c) Organ‐inspired thermal immune current collector|separator design. (d) Bone marrow‐inspired thermal immune unit design. The image of bone is adapted with permission.^[^ ^121^ ^]^ Copyright 2024, the authors.

Furthermore, inspiration can be drawn from the distribution of NK cells within biological organisms to optimize the distribution of thermal immune micro‐nanocapsules, as depicted in **Figure** **9**.

**Thermal immune electrolytes**. NK cells circulate through the bloodstream, distributing to tissues and organs. Under normal conditions, NK cells in blood maintain equilibrium, poised to respond to pathogens or abnormal cells. Inspired by this, the electrolyte, like blood, incorporates thermal immune microcapsules, imparting thermal immunity and enabling real‐time mitigation of TR risks.

**Thermal immune shell**. NK cells are present in the skeletal system of biological organisms, while the battery shell serves a similar supportive function. Therefore, flame retardants can be incorporated into the battery shell, ensuring that they do not come into contact with the internal chemical components of the battery. This design preserves the electrochemical performance while capturing combustion free radicals during TR, thereby preventing exacerbation of the thermal event.

**Thermal immune components**. NK cells are found in organs within biological systems. Similarly, components such as current collectors and separators in batteries can be likened to organs. Thus, thermal immune substances can be integrated into the current collectors and separators, equipping the battery with thermal immune components.

**Thermal immune units**. The bone marrow is the site of NK cell production. Inspired by this, an integrated capsule can be designed and placed between the battery cell and the shell. This integrated capsule would contain thermal immune substances, thus introducing a new thermal immune unit within the battery.

##### Nonflammable Electrolytes Inspired by Free Radical Capture Mechanisms in Nature

**Figure 10:**
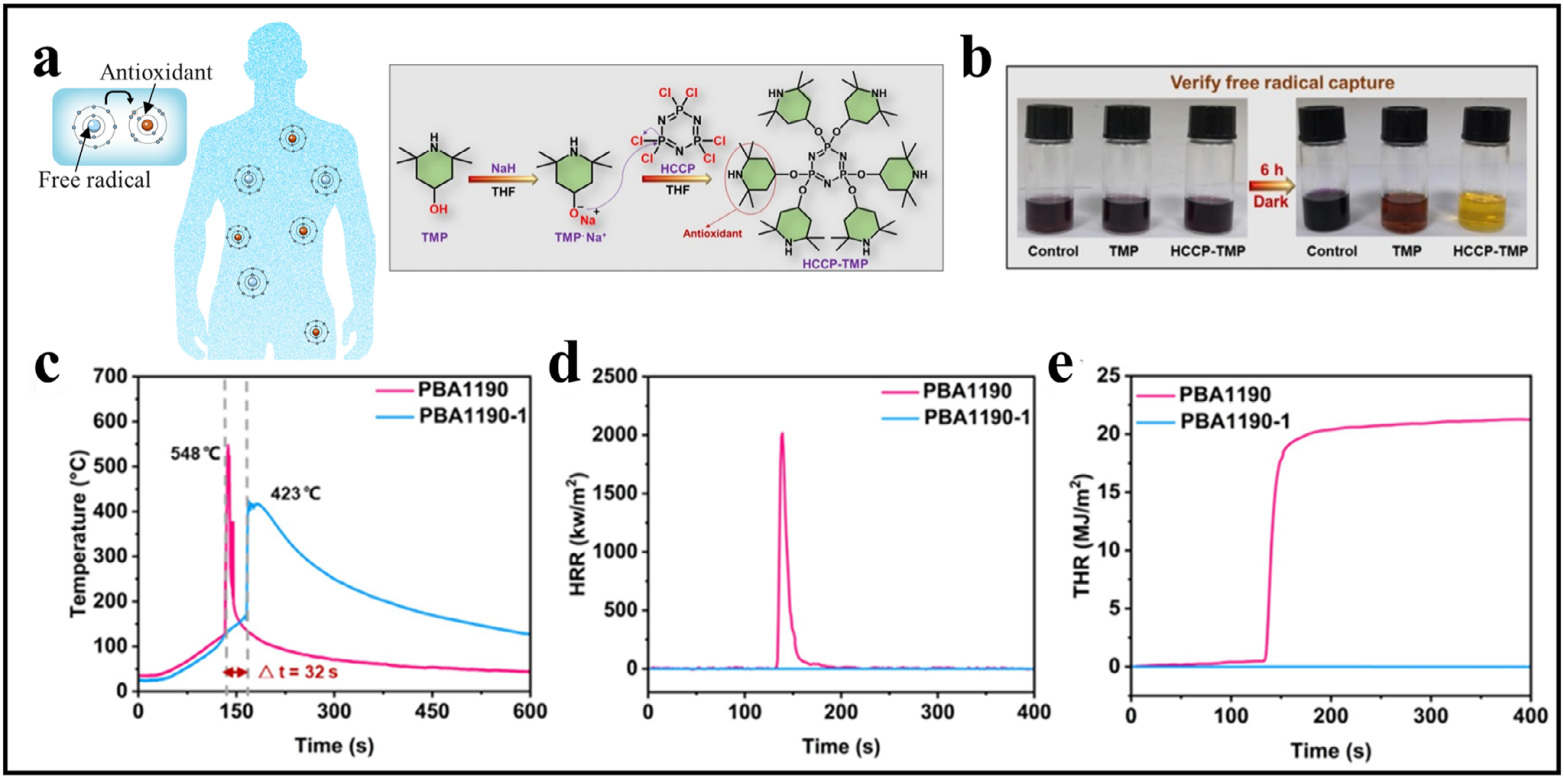
Design and performance testing of flame‐retardant electrolyte additives based on bio‐inspired free radical trapping mechanism. (a) Biomimetic design of a flame‐retardant electrolyte additive inspired by natural free radical trapping mechanisms. The right figure is adapted with permission.^[^ ^79^ ^]^ Copyright 2024, Elsevier Inc. (b) Color change of the solution pre‐ and post‐reaction. Reproduced with permission.^[^ ^79^ ^]^ Copyright 2024, Elsevier Inc. (c) The relationship between surface temperature and time of soft pack batteries. Reproduced with permission.^[^ ^79^ ^]^ Copyright 2024, Elsevier Inc. (d) HRR and (e) THR of the cone calorimeter. Reproduced with permission.^[^ ^79^ ^]^ Copyright 2024, Elsevier Inc.

As illustrated in **Figure** **10**(a), inspired by nature's radical‐capturing mechanisms, Wang et al. developed a biomimetic radical‐capturing electrolyte additive through innovative radical chemistry design, yielding a non‐flammable gel polymer electrolyte (GPE).^[^ ^79^ ^]^ This breakthrough electrolyte effectively addresses multiple bottlenecks in high‐energy‐density batteries.

To evaluate the radical‐capturing ability of HCCP‐TMP (Hexachlorocyclotriphosphazene‐Trimethyl Phosphate), a color‐indicator experiment was conducted, as shown in Figure 10(b). The experiment used 1,1‐diphenyl‐2‐picrylhydrazyl (DPPH) as the radical scavenging indicator, which changes color when reacting with a radical scavenger (DPPH remains purple in its radical form). A control solution of pure DPPH⋅ kept in the dark retained its purple‐black color, while DPPH⋅ solutions containing TMP or HCCP‐TMP turned light yellow after 6 h in darkness, with the HCCP‐TMP group displaying the lightest color. These results indicate that HCCP‐TMP has superior radical‐capturing ability compared to TMP.

Figure 10(c) captures the surface temperature changes of an NCM811||graphite pouch cell before and after TR. When using PBA1190 electrolyte, the pouch cell reached the TR threshold after 133 s of heating, with the surface temperature spiking to 548 °C in just 3 s. In contrast, the pouch cell with PBA1190‐1 electrolyte took 165 s to reach the threshold, delaying TR by 32 s and reaching a maximum surface temperature of only 423 °C. Additional TR tests were conducted on NCM811||PBA1190||graphite and NCM811||PBA1190‐1||graphite pouch cells. At 133 s, when TR began, the NCM811||PBA1190||graphite pouch cell underwent intense heat release accompanied by open flames, which were extinguished by 151 s. This process generated intense heat, with a peak heat release rate (HRR) and total heat release of 2017 kW m^−2^ and 21.2 MJ m^−2^, respectively (Figure 10(d‐e)).

This electrolyte system incorporates HCCP and hindered amine as multifunctional additives, combining HCCP's chemical stability with TMP's radical‐scavenging capabilities. This synergy enables the GPE to exhibit non‐flammability under high‐voltage conditions and effectively suppresses side reactions caused by radicals and singlet oxygen released by high‐nickel cathodes, such as NCM811. Consequently, the risk of electrolyte decomposition and thermal buildup is reduced, enhancing the battery's stability under high‐temperature and high‐pressure conditions. Additionally, the HCCP‐TMP additive improves lithium‐ion transference numbers, decreases polarization effects, and reduces localized current density, thereby inhibiting lithium dendrite growth and lowering short‐circuit risk.

#### 2.2.2 Solid Electrolyte

##### Structured Electrolytes Inspired by Tree Roots

Zinc‐ion batteries have garnered attention due to the inherent advantages of metallic zinc, such as its high theoretical capacity (5854 Ah L^−1^), abundant resources, and safety. However, zinc's low reduction potential (−0.762 V versus SHE) leads to instability in aqueous electrolytes, resulting in hydrogen evolution, corrosion, and the formation of zinc dendrites. These issues reduce zinc utilization, leading to capacity fade and short circuits in the battery. While various strategies, such as hydrogel electrolytes and the “water‐in‐salt” approach, have been proposed to mitigate dendrite formation and side reactions, their effectiveness is limited; they only suppress but do not eliminate these challenges. Therefore, a novel electrolyte is urgently needed that can address these deficiencies while achieving electrochemical performance comparable to aqueous or hydrogel electrolytes.

**Figure 11:**
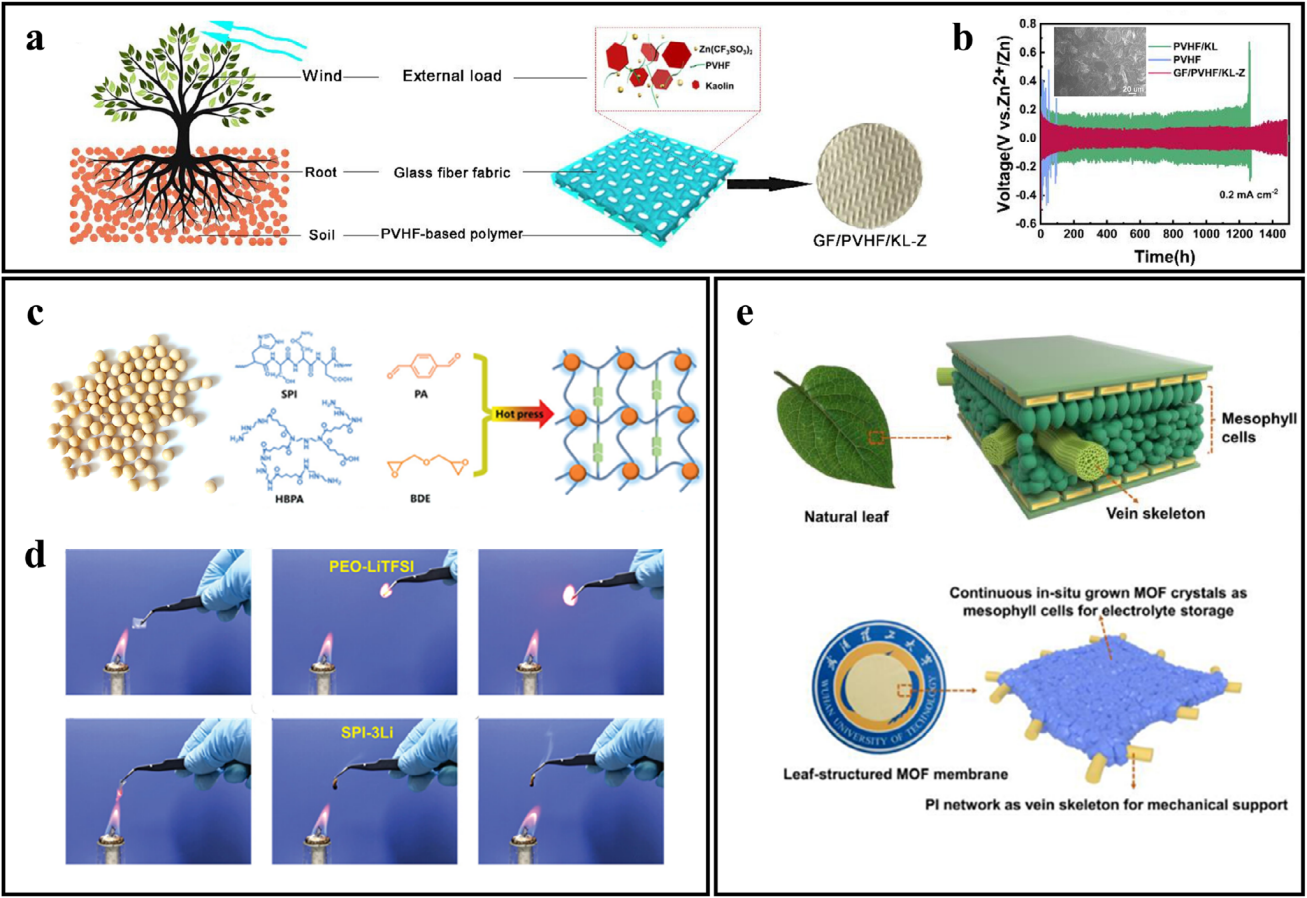
Design and performance study of bio‐inspired structural electrolytes. (a) Analogy between wind‐resistant trees and mechanical load‐resistant structural electrolytes. Reproduced with permission.^[^ ^80^ ^]^ Copyright 2022, Elsevier B.V. (b) Comparison of long – term cycling stability at a current density of 0.2 mA cm^−2^ and SEM image of Zn sheet after extended cycling. Adapted with permission.^[^ ^80^ ^]^ Copyright 2022, Elsevier B.V. (c) Soybeans and fabrication of vitrimer dynamic covalent networks using soy protein isolate. The right figure is reproduced with permission.^[^ ^81^ ^]^ Copyright 2022, Wiley‐VCH GmbH. (d) flame test comparison of PEO|LITFSI film and SPI‐3Li film. Reproduced with permission.^[^ ^81^ ^]^ Copyright 2022, Wiley‐VCH GmbH. (e) Leaf‐inspired design of defect‐free, thin, and robust MOF‐QSEs. Reproduced with permission.^[^ ^82^ ^]^ Copyright 2023, Elsevier B.V.

To address these issues, Liu et al. developed a root‐inspired composite electrolyte based on poly(vinylidene fluoride‐co‐hexafluoropropylene) (PVHF) polymer and glass fiber (GF), as shown in **Figure** **11**(a). The glass fiber fabric mimics the structural support function of roots, while the PVHF polymer resembles the surrounding soil.^[^ ^80^ ^]^ Using a straightforward doctor blade casting technique, they produced a tree‐root‐inspired structured electrolyte (GF|PVHF|KL‐Z) that achieves both high mechanical stiffness and ionic conductivity.

This composite electrolyte combines the high ionic conductivity of the PVHF polymer with the mechanical rigidity of the glass fiber, facilitating rapid Zn^2−^ transport and effectively inhibiting dendrite growth. The battery exhibits outstanding cycling stability, as shown in Figure 11(b), maintaining excellent performance over 1300 h. After prolonged cycling, the Zn surface remains relatively smooth with no significant dendrite formation.

##### Solid Polymer Electrolytes Inspired by Dynamic Crosslinking Network of Soy Protein Isolate

Compared to liquid electrolytes, solid electrolytes offer significant safety advantages. They are typically non‐flammable, which greatly reduces the risk of TR and fire in batteries. Additionally, solid electrolytes effectively prevent leakage issues, thus avoiding potential damage to both the battery and surrounding equipment due to electrolyte spills. Traditional liquid electrolytes, however, face limitations in practical applications due to flammability, the use of volatile organic solvents, and leakage concerns. Solid electrolytes, with their ability to inhibit the formation and growth of lithium dendrites, their non‐flammable nature, and their wider electrochemical window, show substantial potential as replacements for liquid electrolytes. Nonetheless, current solid polymer electrolytes (SPEs) still suffer from drawbacks such as low ionic conductivity, poor mechanical stability, and insufficient thermal stability.

To address these challenges, Gu et al., as illustrated in Figure 11(c), adopted a dynamic imine‐crosslinked network based on soy protein isolate (SPI) as the solid electrolyte matrix, representing a biomimetic utilization of natural materials.^[^ ^81^ ^]^ SPI, as a natural polymer, offers self‐healing properties, echoing the self‐repair capabilities of many biological systems. By incorporating LiTFSI as the ionic conductive filler, the researchers successfully developed an SPI‐based vitrimers material with high mechanical strength and non‐flammable characteristics, demonstrating favorable conductivity and thermal stability.

Using Poly(ethylene oxide)|Lithium bis(trifluoromethanesulfonyl)imide (PEO|LiTFSI) films as a reference sample, the flame‐retardant properties of SPI‐Li‐based SPE films were investigated through flammability testing. In these tests, the SPI‐3Li film (mass ratio of SPI|HBPA (hyperbranched polyamide)|LiTFSI at 1:1:3) exhibited notable flame resistance. As shown in Figure 11(d), the PEO|LiTFSI film ignited immediately and burned vigorously upon contact with a flame. In stark contrast, the SPI‐3Li film deformed at high temperatures but did not catch fire even after several seconds of flame exposure. This study highlights the enhanced application potential of solid polymer electrolytes in LIBs, addressing issues such as flammability and low mechanical strength associated with traditional electrolytes. Moreover, the dynamic crosslinked network in SPI provides recyclability and reprocessing capabilities, supporting sustainable manufacturing practices.

##### Metal–Organic Framework‐Based Solid Electrolytes Inspired by Leaf Structure

In lithium metal battery research, dendrite growth poses a critical issue by causing uneven lithium‐ion distribution on the lithium metal surface, leading to short circuits and other safety risks. Traditional separator designs struggle to regulate lithium‐ion deposition effectively due to uneven pore distribution, which in turn promotes dendrite formation. Additionally, metal‐organic framework‐based solid electrolytes (MOF‐QSE) tend to be inherently brittle and have poor compatibility with polymer binders, resulting in structural instability during battery assembly, increased weight, and reduced energy density. To address these issues, Du et al. (see Figure 11(e)) designed a novel leaf‐inspired MOF‐QSE, drawing inspiration from the structure and function of natural leaves.^[^ ^82^ ^]^ Leaves consist primarily of the epidermis, mesophyll, and vein skeleton. Mesophyll cells are the site of photosynthesis, where light energy is converted into chemical energy stored in organic compounds, which are then transported to various parts of the plant. The vein skeleton provides structural support, enabling leaves to withstand harsh conditions like wind and rain. This serves as an ideal reference for developing a defect‐free, thin, and robust MOF‐QSE.

To replicate leaf structure, a thin yet strong polyimide (PI) fiber network with high electrochemical stability was introduced into the MOF‐QSE as the vein skeleton, providing mechanical support. This PI fiber network, with a high porosity of 83%, creates ample space for MOF crystals to grow, allowing high‐quality MOF crystal loading within the leaf‐inspired MOF membrane. MOF crystals grow in situ on the PI framework, forming a continuous and defect‐free MOF film that acts as “mesophyll cells” for electrolyte storage and transport. Finally, a liquid electrolyte is introduced into the micropores of the MOF crystals, simulating the “blood” of the battery.

By growing MOF crystals in situ on the PI fiber network, researchers successfully formed a continuous and defect‐free MOF film, ensuring even lithium‐ion distribution on the lithium metal surface and effectively suppressing dendrite growth. Furthermore, the controllable thickness of this novel MOF‐QSE significantly reduces ion transport resistance, thereby enhancing the battery's energy density.

### 2.3 Separators

Commercial PE separators currently in use tend to shrink and soften at high temperatures and are highly susceptible to being punctured by lithium dendrites, which can lead to short circuits, TR, and even battery explosions. These challenges underscore the importance of improving the thermal stability and mechanical properties of battery separators. While the reinforcement of separators with inorganic ceramic materials has improved thermal stability, there remains a significant mismatch in mechanical properties between the ceramic layer and the PE substrate. This non‐uniform structure risks ceramic layer detachment increases lithium‐ion channel resistance, and can ultimately cause uneven lithium‐ion flow, which fosters lithium dendrite growth. Additionally, such approaches often involve complex fabrication processes and high costs, posing challenges for large‐scale production.^[^ ^50^, ^83^, ^84^ ^]^

#### 2.3.1 LIB Separators Inspired by Mammalian Enamel Structure

**Figure 12:**
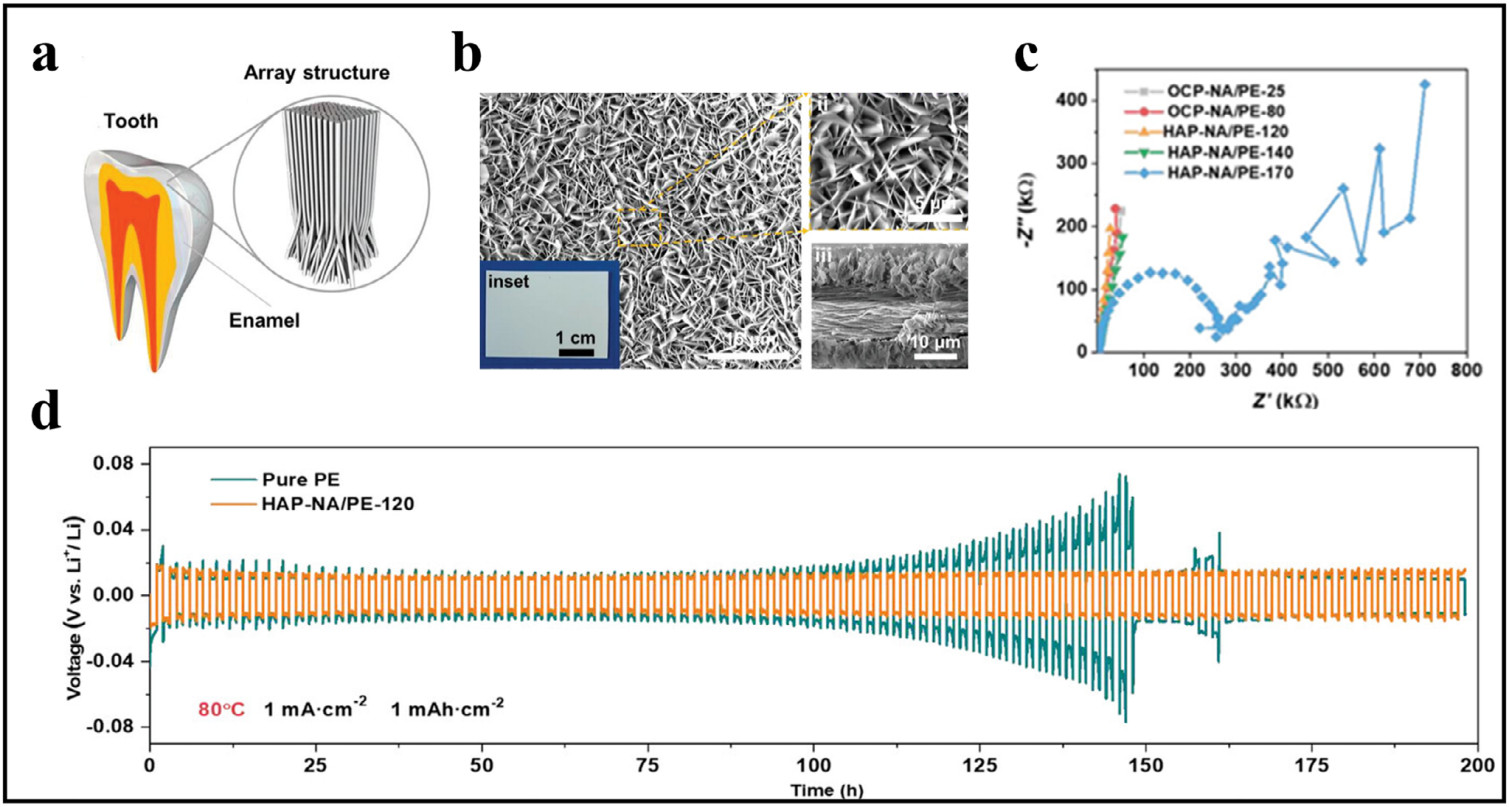
The design inspiration and partial performance characterization of the enamel‐inspired HAP‐NA|PE nanocomposite separator. (a) Diagram illustrating the well‐organized HAP nanorod array structure in the enamel of mammalian teeth. Reproduced with permission.^[^ ^85^ ^]^ Copyright 2023, Wiley‐VCH GmbH. (b) Surface and cross‐sectional morphology of OCP‐NA|PE‐25 at varying magnifications. Reproduced with permission.^[^ ^85^ ^]^ Copyright 2023, Wiley‐VCH GmbH. (c) Nyquist plots for stainless steel/stainless steel cells using different nanocomposite separators at 25 °C. Reproduced with permission.^[^ ^85^ ^]^ Copyright 2023, Wiley‐VCH GmbH. (d) Long‐term cycling stability of Li–Li cells with PE and HAP‐NA|PE‐120 separators at 80 °C. Reproduced with permission.^[^ ^85^ ^]^ Copyright 2023, Wiley‐VCH GmbH.

Yue et al. drew inspiration from the structure of mammalian tooth enamel, in which highly ordered hydroxyapatite (HAP) nanorods interlock with an organic matrix, creating a strong mechanical protective structure.^[^ ^85^ ^]^ Mimicking this enamel microstructure, they successfully designed a 3D interlocking HAP‐nanosheet array (NA|PE composite membrane structure with mutually supportive elements, which greatly enhances the mechanical strength and thermal stability of the separator (see **Figure** **12**(a)). Figure 12(b) illustrates the surface and cross‐section morphologies of octa‐calcium phosphate (OCP)‐NA|PE‐25 under different scales.

As shown in Figure 12(c), the impedance of stainless steel/stainless steel cells with the nanocomposite separator significantly increases when heated above 140 °C, indicating effective thermal shutdown and ionic channel closure. This response can effectively interrupt electrochemical reactions, thereby preventing severe TR under mechanical or thermal abuse conditions.

In terms of cycling stability and lifespan, Figure 12(d) demonstrates that the use of the nanocomposite separator in Li|HAP‐NA‐120|Li cells enhances performance substantially at room temperature. Even at 80 °C, Li|HAP‐NA|PE|Li cells exhibit longer and stable cycling performance. In contrast, Li‐Li cells with standard PE separators in ether electrolytes exhibit an increase in overpotential from 30 to 70 mV, primarily due to depletion of the ether electrolyte and the concomitant growth of interfacial resistance associated with dead‐Li accumulation. By comparison, Li–Li cells with HAP‐NA|PE‐120 separators maintain an overpotential as low as ≈20 mV for up to 200 h, demonstrating superior cycling stability. In addition, the HAP‐NA layer improves the separator's flame retardancy, enhancing cell fire safety.

Compared to other separators, the HAP‐NA|PE separator excels in lithium dendrite suppression, high‐temperature stability, and thermal shutdown safety. This biomimetic design addresses the detachment issue found in traditional ceramic coatings, preventing lithium‐ion channel blockage while inhibiting dendrite growth.

### 2.4 Anodes

The anode material critically affects LIB safety. Graphite is stable but may form dendrites under fast charging or low temperatures. Silicon suffers from volume expansion, increasing dendrite risk, while lithium titanate offers high thermal stability and minimal dendrite formation. Metallic lithium provides high energy but is highly unsafe.^[^ ^86^ ^]^ Multivalent anodes like zinc are safer but face electrolyte corrosion. To enhance anode performance, Anode coatings—especially hydrophilic or conductive ones—can suppress dendrite growth, reduce interfacial reactions, improve heat dissipation, protect against corrosion, and enhance overall battery stability and safety.^[^ ^87^, ^88^, ^89^ ^]^

#### 2.4.1 Artificial Interface Layers for Lithium Metal Anodes Inspired by Fish Scales

Lithium‐sulfur batteries offer high energy density and eco‐friendly sulfur but face commercialization challenges due to lithium dendrite formation and polysulfide shuttling, causing short cycle life, low Coulombic efficiency, and safety risks. Polysulfide diffusion to the anode triggers side reactions, uneven lithium deposition, and dendrite growth, while fragile SEI layers accelerate degradation and increase internal resistance, making a stable lithium metal anode interface crucial.

**Figure 13:**
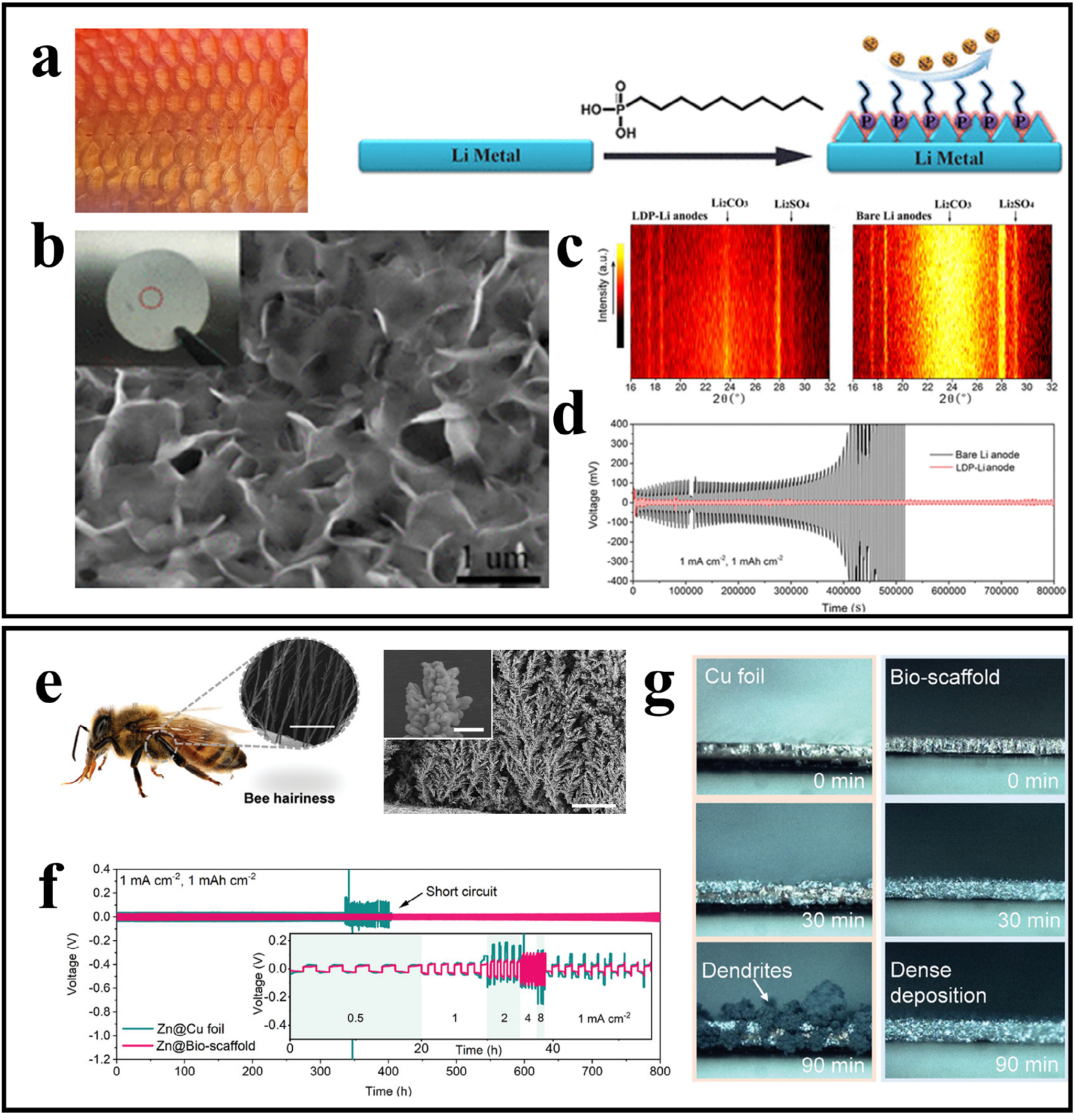
Application and electrochemical performance of bio‐inspired design in lithium|zinc metal anodes. (a) Fish scales and chematic illustration showing the design and impact of the LDP layer on the Li surface during cycling. The right figure is reproduced with permission.^[^ ^90^ ^]^ Copyright 2018, American Chemical Society. (b) SEM images of LDP‐Li surface. Reproduced with permission.^[^ ^90^ ^]^ Copyright 2018, American Chemical Society. (c) In situ XRD patterns of LDP‐Li and bare Li anodes during the first charge/discharge cycle. Reproduced with permission.^[^ ^90^ ^]^ Copyright 2018, American Chemical Society. (d) Voltage of symmetric lithium battery at 1 mA cm^−2^. Reproduced with permission.^[^ ^90^ ^]^ Copyright 2018, American Chemical Society. (e) Optical image of a pinned honeybee, with an inset SEM image of bee hairiness. Adjacent are the 3D fractal Cu array and its cross‐sectional SEM. Reproduced with permission.^[^ ^91^ ^]^ Copyright 2022, Wiley‐VCH GmbH. (f) Voltage profiles of symmetric cells with Zn@Cu foil and Zn@Bio‐scaffold electrodes at 1 mA cm^−2^ and 1 mAh cm^−2^ capacity, with rate performance at various current densities shown in the inset. Reproduced with permission.^[^ ^91^ ^]^ Copyright 2022, Wiley‐VCH GmbH. (g) Schematic of multivalent metal plating/stripping on the Bio‐scaffold. Reproduced with permission.^[^ ^91^ ^]^ Copyright 2022, Wiley‐VCH GmbH.

Fish scales consist mainly of proteins with a hydrophilic calcium phosphate framework covered by a thin mucus layer, allowing fish to swim freely even in oil‐polluted waters without fouling their skin. Inspired by this hydrophobic property, Shen et al. constructed a scale‐like polysulfide artificial interface layer on the lithium metal anode surface, as shown in **Figure** **13**(a).^[^ ^90^ ^]^ The lithium decylphosphonate (LDP)‐Li anode, seen in Figure 13(b), exhibits a 3D scale structure, and Figure 13(c) presents in situ XRD patterns collected at 0.1 C current density, where Li_2_SO_4_ and Li_2_CO_3_ diffraction peaks are notably weaker in LDP‐Li anodes compared to bare lithium, indicating that the LDP layer effectively reduces the highly active, irreversible reactions between the organic electrolyte and lithium metal.

The study further evaluated the protective effect of the LDP coating using symmetric cells assembled with a 1 M LiPF_6_ electrolyte in EC: DMC. Figure 13(d) shows the long‐term overpotential curve at 1 mA cm^−2^ current density and 1 mAh cm^−2^ cycling capacity. For the bare lithium cell, the overpotential continuously increases after ≈50 cycles, eventually rising sharply above 200 mV due to SEI insulation and breakdown from continuous electrolyte reactions, which may hinder ion transport at the interface. In contrast, the LDP‐Li anode‐based cell exhibits a flat, consistent voltage platform with overpotential remaining below 20 mV for 200 h, demonstrating enhanced stability compared to bare lithium.^[^ ^90^ ^]^

Similar to how the unique structure of fish scales prevents oil contamination, the artificial interface layer repels polysulfides effectively, reducing side reactions and promoting uniform lithium deposition to inhibit dendrite formation. This biomimetic design maintains the structural integrity of the artificial interface layer throughout cycling, preventing SEI layer fracture and repair cycles. This improvement increases the battery's cycle life and Coulombic efficiency (approaching 99%), with a high capacity of 1000 mAh/g that remains stable after 200 cycles.^[^ ^90^ ^]^

#### 2.4.2 Bio‐Inspired Bee Scaffold for Multivalent Metal Batteries

Multivalent metal batteries, such as zinc and aluminum batteries, are promising for large‐scale energy storage due to their low cost, safety, and high capacity. However, challenges like the incompatibility of metal anodes with electrolytes, especially in aqueous zinc batteries where hydrogen evolution and zinc corrosion occur, as well as corrosion from high‐chloride electrolytes in aluminum batteries, significantly hinder their cycle life and safety. Surface defects on these anodes cause uneven electric field distribution, leading to uncontrolled dendrite growth, which heightens safety risks and accelerates interface side reactions, ultimately degrading cycling performance.

Existing approaches, such as electrolyte engineering and anode coatings, struggle to achieve effective atomic‐level interface modifications. While 3D scaffolds can suppress dendrite growth, they increase ion transport distances and intensify interface side reactions. Inspired by the natural structure of bee legs, which are covered with large‐surface‐area, parallel, fractal hair structures for effective pollen collection, Zhang et al. developed a bio‐inspired scaffold (Bio‐scaffold) for multivalent metal anodes, specifically to control interface behavior and inhibit dendrite growth. As shown in Figure 13(e), this scaffold features a 3D fractal Cu array with parallel Cu branches, which are coated with a CaTiO_3_ (CTO)‐based protective layer.^[^ ^91^ ^]^

A symmetric Zn@Bio‐scaffold||Zn@Bio‐scaffold cell was assembled to test long‐term constant current cycling performance at 1 mA/cm^2^. Comparative cells using Zn@Cu foil and Zn@3D Cu array electrodes were also tested. The Zn@Cu foil cell exhibited voltage lag (≈39 mV) at 404 h, followed by a sudden drop to 0 V, indicating dendrite‐induced shorting (Figure 13(f)). In contrast, the Zn@3D Cu array cell had an average overpotential of 29 mV but saw a rapid increase at 342 h due to side reactions. However, the Zn@Bio‐scaffold cell displayed a stable and flat voltage platform, with a reduced lag of ≈26 mV, maintaining stability over 800 h without noticeable changes (Figure 13(f)).

The Zn@Bio‐scaffold electrode also demonstrated excellent rate performance: as current density increased from 0.5 to 8 mA cm^−2^, voltage lag was maintained at 14, 26, 51, 92, and 109 mV, respectively, significantly lower than Zn@Cu foil cells (inset in Figure 13(f)). This indicates that the bio‐inspired scaffold's parallel structure effectively shortens ion transport distance, reduces local current density, enables fast zinc‐ion diffusion, and regulates zinc deposition at high current densities.

To further observe morphological changes during zinc plating, a transparent Zn||Cu asymmetric cell was monitored in situ at 5 mA/cm^2^. After 90 min of plating, the Zn deposited on bare Cu foil exhibited a highly porous, dendritic morphology (Figure 13(g) left), whereas the bio‐inspired scaffold produced a compact deposition without dendrites or bubbles (Figure 13(g) right).

In summary, the bio‐inspired design, with its parallel copper branches, enhances plating/stripping kinetics and improves electrolyte wettability, reducing local current density and effectively suppressing dendrite formation. The CaTiO_3_‐based protective layer prevents interface side reactions and corrosion, while its high dielectric constant controls multivalent ion flux for uniform nucleation. This scaffold allows for dendrite‐free plating/stripping of zinc and aluminum, achieving Coulombic efficiencies of 99.83% (zinc) and 99.81% (aluminum). It enables 4000 cycles at 5 mA cm^−2^ for zinc batteries and 1500 cycles for aluminum without dendrite growth. Zn||V_10_O_24_·12H_2_O and Al||graphite batteries using this bio‐inspired scaffold exhibit higher specific capacities and longer cycle lives.^[^ ^91^ ^]^

## 3 System Safety Biomimetic Design for Batteries

### 3.1 Thermal Management Designs

Battery thermal management is essential for safety and performance, maintaining cells within 20–40 °C and ensuring uniform temperature distribution to prevent TR. Conventional methods—air, liquid, PCM, and hybrid cooling—can regulate temperature but suffer from local overheating, high energy consumption, and poor responsiveness to sudden TR events. With high‐energy‐density batteries, these limitations pose serious safety challenges. Biomimetic design offers innovative solutions by mimicking natural thermal regulation mechanisms, such as plant leaves or desert lizards, to enhance heat dissipation, reduce energy use, and improve temperature uniformity. Applying such principles to existing cooling strategies has advanced battery technology and opened new avenues for safer, more efficient thermal management systems.^[^ ^92^, ^93^, ^94^ ^]^

#### 3.1.1 Air Cooling Systems

Air cooling systems offer simplicity, low cost, no leakage risk, and easy installation, but their low thermal conductivity limits cooling capacity, leading to uneven temperature distribution, especially under high discharge rates, dense packing, or fast charging. To address this, research focuses on optimizing airflow, battery layout, and cooling channel design, as well as adding auxiliary components to enhance turbulence and heat transfer area. Incorporating heat exchangers can further improve efficiency, ensuring safer and more reliable battery operation under demanding conditions.

##### Biomimetic Radiator Inspired by Cranial Structure of Crodocodiles 

In nature, many organisms have evolved specialized structures to enhance thermal exchange efficiency with their environments, adapting to different conditions. These biomimetic structures have inspired the optimization of heat exchange devices, providing new insights for improving air cooling systems and advancing their application in battery thermal management.

**Figure 14:**
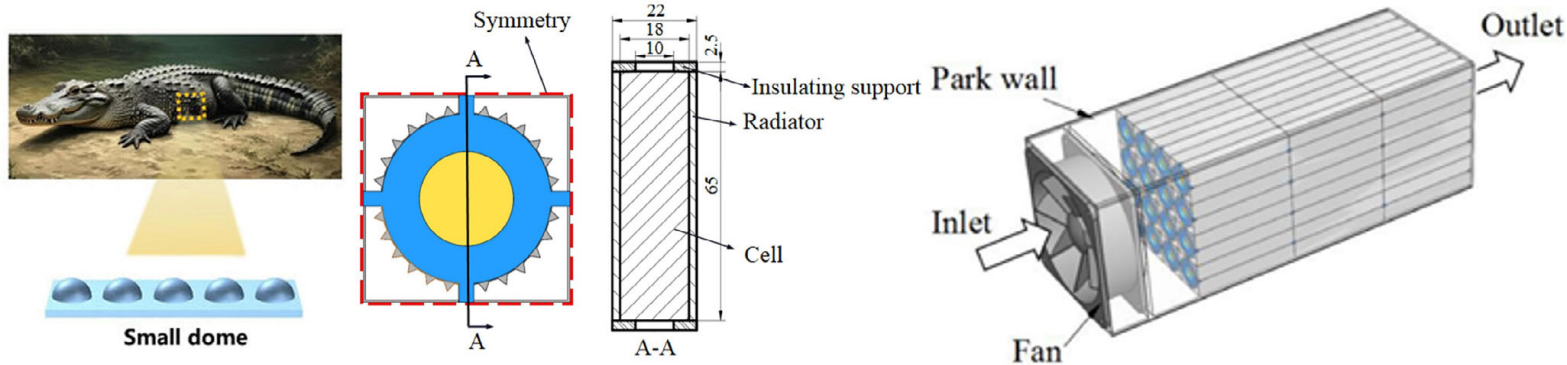
A bionic heat sink designed for axial air cooling of cylindrical battery modules inspired by the crocodile skull and bone structure. The left panel shows the characteristic features of crocodile skin. Adapted with permission.^[^ ^118^ ^]^ Copyright 2025, Wiley‐VCH GmbH. The middle panel illustrates a cylindrical battery cooling structure inspired by crocodile skin. The right panel presents a battery pack cooling system. The middle and right images are adapted with permission.^[^ ^95^ ^]^ Copyright 2020, Elsevier Ltd.

As shown in **Figure** **14**, Inspired by the cranial structure of crocodiles and their bone composition, Yang et al. designed a biomimetic heat sink specifically for the axial air cooling of 18 650 cylindrical battery modules.^[^ ^95^ ^]^ This heat sink can be applied to battery packs without altering the arrangement of the battery cells. Experimental results indicate that the optimized battery module achieved reductions of 8.1% in temperature difference and 15.54% in power consumption compared to traditional heat sinks. When the inlet air velocity exceeds 0.8 m/s, the maximum battery temperature and temperature difference can be maintained within 313 and 5 K, respectively.

This air‐cooled battery thermal management system (BTMS) design allows the heat generated by the battery to be effectively transferred through solid conduction to the heat sink, where cooling air disperses the heat, thus enabling efficient temperature control. This approach not only enhances cooling performance but also contributes to the overall safety and longevity of battery systems, particularly in high‐demand applications.

#### 3.1.2 PCM Cooling Systems

PCMs offer high latent heat storage and excellent temperature uniformity, making them promising for battery thermal management. By absorbing excess heat during melting and releasing it upon solidification, PCMs stabilize system temperatures and prevent overheating. However, challenges such as added volume and weight, high costs, and limited cooling capacity under high discharge rates hinder practical adoption. Organic PCMs (e.g., paraffin, fatty acids) provide good stability but are flammable and insufficient for high‐power applications, while enhancements like fins or metal foams improve conductivity but raise costs. Current research thus focuses on novel PCM formulations, composites, and structural designs to balance performance, safety, and cost for large‐scale battery applications.

##### Phase Change Material Cooling Structure Inspired by Serpentine Design

Composite Phase Change Materials (CPCMs) are innovative materials that combine phase change materials with other substances, such as thermal conductive fillers or reinforcement materials. The primary purpose of adding these fillers is to enhance the thermal conductivity, mechanical strength, and stability of the phase change material, thereby improving the overall performance of the cooling system.

**Figure 15:**
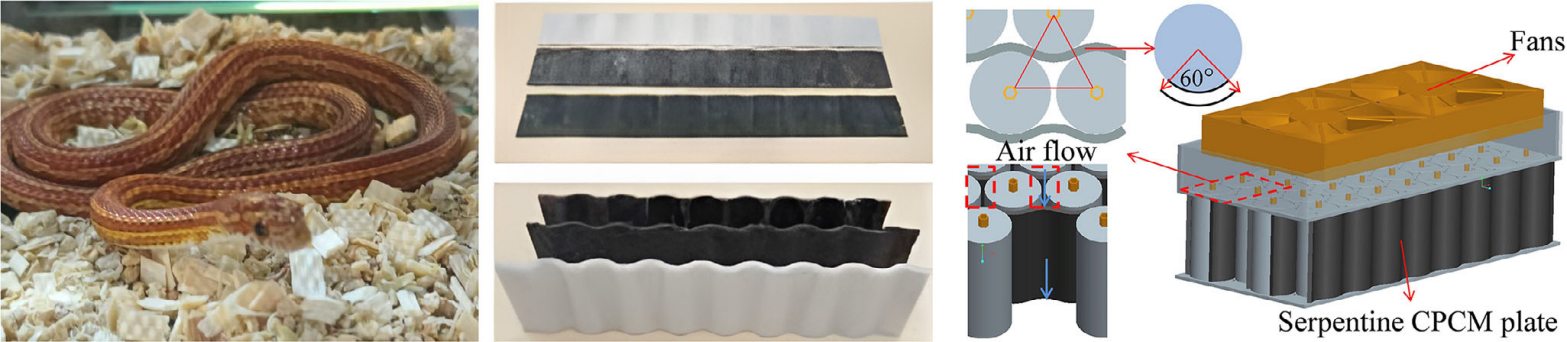
Composite phase change material panel with a serpentine structure. The left panel shows a snake. The middle panel presents the physical structure of biomimetic snake‐shaped cooling fins. The right panel illustrates a battery cooling system. The middle and right images are adapted  with permission.^[^ ^96^ ^]^ Copyright 2020, Elsevier B.V.

Recent advancements have been made in PCM cooling structures, as shown in **Figure** **15**, as highlighted by research conducted by Lv et al. They developed a novel cooling solution using a serpentine composite phase change material (S‐CPCM) plate to replace traditional block‐shaped composite phase change material (B‐CPCM) modules.^[^ ^96^ ^]^ The results from multiple charge and discharge cycles demonstrated that the maximum temperature of the S‐CPCM module was significantly lower than that of the B‐CPCM module (51.9 °C versus 54.2 °C), indicating its superior heat dissipation performance.

The S‐CPCM cooling structure achieves efficient thermal regulation while reducing CPCM usage by ≈70%, thereby increasing battery module energy density by 13.8 Wh kg^−1^ and enabling a lighter, more efficient design. Unlike traditional block‐shaped CPCM modules, S‐CPCM plates offer superior shape stability and conform closely to cylindrical cells, minimizing weight without sacrificing cooling performance. These advantages highlight the potential of CPCMs to improve both safety and performance in battery systems requiring effective thermal management.^[^ ^96^ ^]^

#### 3.1.3 Liquid Cooling Systems

Liquid cooling systems, with superior thermal conductivity and heat capacity, offer more stable and continuous heat dissipation than air or PCM cooling, making them the mainstream solution for high‐power batteries and electric vehicles. Research mainly optimizes cooling channel designs (e.g., S‐type, loop‐type) and balances flow rate, heat flux, and pressure drop, with biomimetic approaches offering further gains in efficiency and energy savings. Liquid cooling can be direct or indirect: immersion cooling provides rapid, uniform heat removal and simplifies design but faces challenges in coolant compatibility, sealing, leakage risk, system complexity, and added weight, limiting its application in space‐ and weight‐sensitive systems.

##### The Flow Guides with Fish‐Shaped Holes is Inspired by the Streamlined Structure of Fish

**Figure 16:**
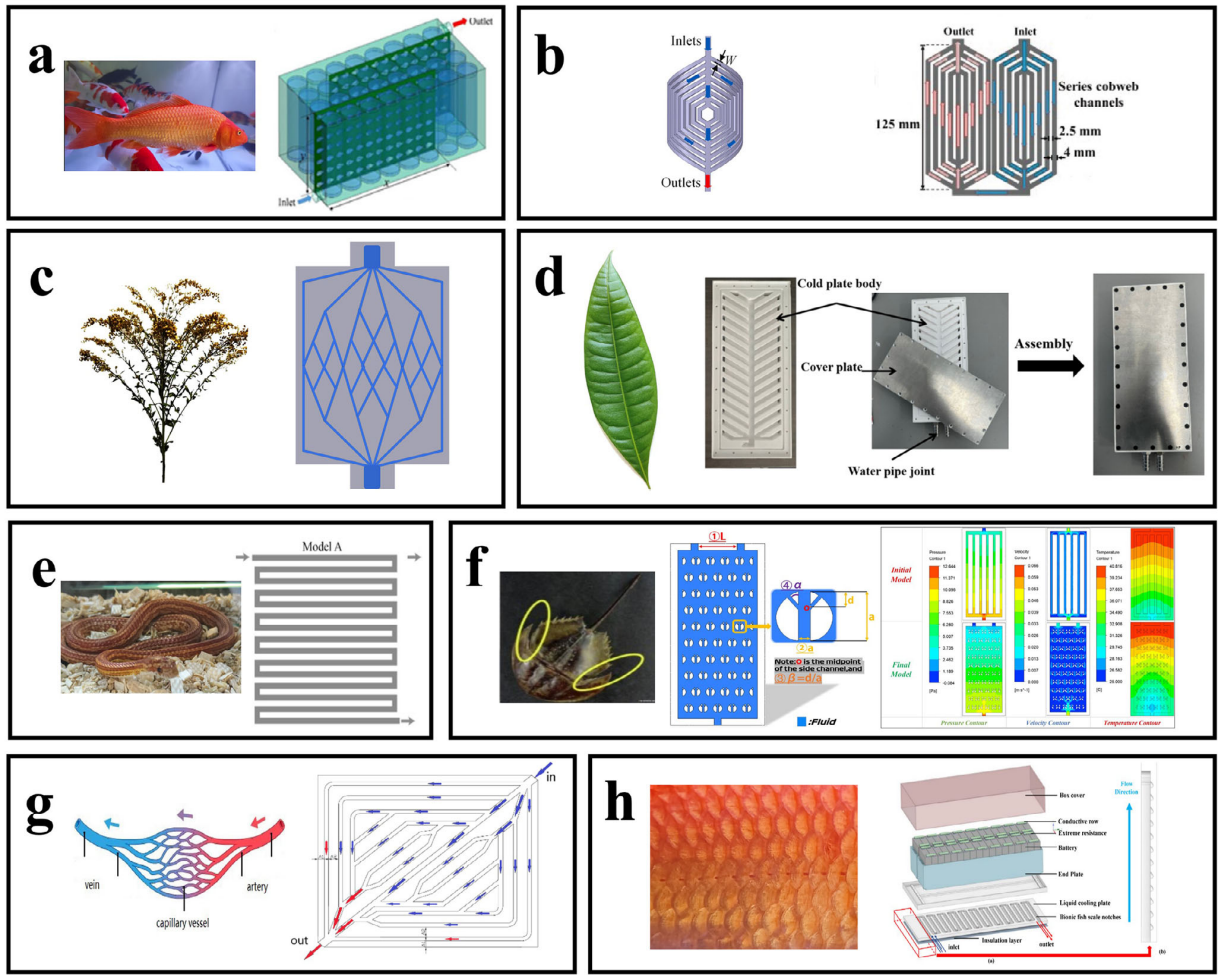
Application and optimization of bio‐inspired design in liquid cooling BTMS. (a) Flow guides with fish‐shaped holes, inspired by the streamlined bodies of fish. The right image is reproduced   with permission.^[^ ^97^ ^]^ Copyright 2023, Elsevier Ltd. (b) A heat exchanger inspired by the web‐like structure of spider silk. Adapted with permission.^[^ ^98^ ^]^ Copyright 2023, Elsevier Ltd. Reproduced with permission.^[^ ^99^ ^]^ Copyright 2024, Elsevier Ltd. (c) A channel cold plate modeled after tree‐like structures. (d) Leaf‐inspired liquid cold plate design. The right image is reproduced with permission.^[^ ^102^ ^]^ Copyright 2024, Elsevier Ltd. (e) A snake and a cooling plate with a serpentine design. The right image is adapted with permission.^[^ ^106^ ^]^ Copyright 2009, Elsevier B.V. (f) A  cooling plate with bionic fins. Adapted and reproduced with permission.^[^ ^108^ ^]^ Copyright 2023, Elsevier Ltd. (g) A bionic cooling plate inspired by human blood vessels. Reproduced with permission.^[^ ^109^ ^]^ Copyright 2022, MDPI. (h) Optimized channel design inspired by fish scales. The right image is reproduced with permission.^[^ ^111^ ^]^ Copyright 2023, MDPI.

As shown in **Figure** **16**(a), Gao et al. drew inspiration from the streamlined structure of fish, designing a flow guide with fish‐shaped holes to optimize the flow distribution within battery modules and reduce pump power consumption.^[^ ^97^ ^]^ This design combines conformal mapping techniques with biomimetic principles to create fish‐shaped perforations on the flow guide. At a mass flow rate of 0.00273 kg/s, the fish‐shaped hole design achieved a 12.2% reduction in maximum battery temperature under a 3C discharge rate compared to conventional flow guides. Additionally, the pump power consumption was minimized with the fish‐shaped flow guide, indicating improved cooling performance alongside reduced energy consumption.^[^ ^97^ ^]^ As the discharge rate increased, the temperature uniformity of batteries using the fish‐shaped flow guide improved significantly; at a 3C rate, the temperature difference in the conventional guide was 5.12 °C, whereas it was only 4.38 °C with the biomimetic flow guide. This innovative design not only enhances the efficient heat dissipation capabilities of direct liquid cooling systems but also addresses some inherent limitations by reducing energy consumption and increasing temperature uniformity, providing a novel approach for optimizing direct liquid cooling technology.

In contrast, indirect cooling involves indirect contact with the battery surface through a cold plate or cooling channels. However, indirect cooling efficiency is limited by the presence of air gaps between the battery and the cold plate, which act as insulators and lower thermal transfer efficiency. To address this, thermal grease or epoxy resin is typically applied to eliminate these gaps, although this approach increases the system's complexity, weight, and cost.

##### Heat Exchanger/Cold Plate Inspired by Spider Web Structure

Spider web structures in nature are known for their unique distribution uniformity and efficient transmission properties. Inspired by this structure, as shown in Figure 16(b), Xiong et al. developed an innovative heat exchanger that leverages its large surface area and high convective heat transfer coefficient to effectively maintain battery temperature with minimal power consumption.^[^ ^98^ ^]^ At a flow rate of 0.4 g s^−1^, the heat exchanger achieves a maximum temperature (T_max_) of 302.972 K, a temperature difference (ΔT) of 3.858 K, and a pressure drop (ΔP) of 22.75 Pa. When compared to honeycomb and traditional spiral structures with the same volume fraction and geometric parameters, the spider web‐inspired design demonstrates superior thermal and flow properties.^[^ ^98^ ^]^

Also inspired by spider web structures, as shown in Figure 16(b), Xie et al. proposed a novel BTMS featuring a new BC‐LCP design with a differential velocity distribution strategy to address low temperature uniformity.^[^ ^99^ ^]^

Wang et al. were similarly inspired by the spider web structure in nature, proposing a cold plate with biomimetic spider web channels.^[^ ^100^ ^]^ Focusing on the heat generation characteristics of soft‐pack LIBs at a 12C maximum discharge rate, they used an orthogonal experimental design to analyze the cooling performance of LIB channels modeled after spider webs. Through numerical simulations, they investigated the impact of various spider web channel parameters on the cooling performance of the cold plate.

##### Cold Plates Inspired by Plant Structures

The vascular system of plant leaves is highly optimized for fluid transport and heat dissipation, enabling these natural structures to effectively adapt to complex environmental conditions while ensuring efficient energy and nutrient delivery. Such characteristics make them particularly well‐suited for liquid cooling systems, especially in managing heat dissipation for large‐scale battery systems.

As shown in Figure 16(c), Zhan et al. proposed a novel tree‐structured channel cold plate.^[^ ^101^ ^]^ Through an orthogonal experimental design, they investigated the effects of inlet flow rate, number of inlet channels, channel width, and hierarchical ratio on battery T_max_, standard temperature difference (T_σ_), and ΔP. Using the Non‐dominated Sorting Genetic Algorithm II (NSGA‐II), an optimized solution set was obtained. Results indicate that, under optimal parameters, the developed cold plate reduced T_max_ by up to 13.94%, Tσ by up to 52.94%, and ΔP by up to 61.5% compared to a traditional straight‐through cold plate. The comprehensive index PEC significantly improved by 89%.^[^ ^101^ ^]^

As shown in Figure 16(d), Chen et al. introduced an innovative cold plate design with dual‐layer biomimetic leaf vein channels.^[^ ^102^ ^]^ The main design concept mimics the primary and secondary veins in leaves that provide moisture and nutrients, resulting in a biomimetic leaf vein structure with main and branch channels. Results indicate that battery modules with dual‐layer leaf vein channels exhibit superior performance in terms of maximum temperature, temperature difference, and inlet/outlet pressure drop compared to traditional channels. This innovative channel design combines the advantage of lower maximum temperatures seen in serpentine channels with the reduced inlet/outlet pressure drop characteristic of parallel straight channels.

Inspired by leaf structures, Zhang et al. developed a biomimetic cooling plate structure, showing that the thermal performance and system energy consumption of the cold plate were primarily influenced by structural factors such as coolant mass flow rate, bifurcation channel angle, channel width, channel height, and the distance between bifurcation channels.^[^ ^103^ ^]^ Using these six parameters as design variables, they optimized the total thermal resistance (TTR) and ΔP of the liquid cooling plate through a Neighborhood Cultivation Genetic Algorithm. Compared with the pre‐optimized model, the TTR and ΔP of the optimized cold plate were reduced by 0.2409 K/W and 8.7371 Pa, respectively.^[^ ^103^ ^]^

Liu et al. developed a liquid cooling plate with biomimetic leaf vein branches (BLVB) embedded with pouch LIBs.^[^ ^104^ ^]^ Using the orthogonal range test method, they analyzed the effects of multi‐parameter coupling for inlet flow rate, channel width, channel angle, and channel number on the T_max_, maximum temperature difference (ΔT_max_), and average pressure drop (ΔP_avg_) of the battery. During 3C discharge, T_max_ was controlled within 33.34 °C. After optimizing the cooling plate parameters, the best results were T_max_ = 30.31 °C, ΔT_max_ = 2.78 °C, and ΔP_avg_ = 0.50 kPa. When M = 0.10 m/s, the optimal BLVB channel reduced T_max_ by 0.23 °C and 1.12 °C, ΔT_max_ by 0.28 °C and 1.64 °C, and ΔP_avg_ by 65.56% and 8.77%, respectively.^[^ ^104^ ^]^

As shown in Figure 16(d), Tang et al. proposed a new biomimetic cooling plate inspired by the leaf vein structure of plantain.^[^ ^105^ ^]^ Results demonstrated that a single‐inlet, dual‐outlet leaf vein channel cooling plate exhibited excellent comprehensive performance. The reduced angle of the main inlet channel decreased pressure drop by 43.55% but did not improve temperature uniformity; the maximum temperature difference increased by 0.11 °C. An increased number of branch channels and a smaller branch channel angle enhanced BTMS cooling performance, while increased branch channel width significantly reduced pressure drop.

##### Cold Plates Inspired by Snake Structures

In current liquid cooling plate designs, S‐shaped circuits are widely used. This S‐shaped structure, resembling a snake in form, draws inspiration from the physiological structure and movement characteristics of snakes, thereby enhancing the design of liquid cooling plates.

Yu et al. developed six types of serpentine channel designs (as shown in Figure16(e) )—including traditional serpentine flow fields, four MPSFF designs, and a spiral flow field—as coolant flow channels for cooling plates.^[^ ^106^ ^]^ Computational Fluid Dynamics (CFD) was employed to accurately simulate fluid flow and heat transfer effects. The cooling plate performance was evaluated based on maximum surface temperature and the temperature uniformity index obtained from CFD calculations.

Sheng et al. developed an LCP with dual inlets and outlets in a serpentine channel design to control adverse temperature distribution in battery modules operating at high current.^[^ ^107^ ^]^ A dimensionless analysis of the LCP's hydraulic performance was conducted to help analyze the thermal management operating costs of the developed system.

The biomimetic serpentine design offers numerous insights for enhancing the S‐shaped circuit structure of liquid cooling plates, especially in fluid dynamics optimization, heat exchange efficiency, structural flexibility, and adaptability. The snake's streamlined body and flexible adaptability contribute to designing liquid cooling plate systems that are more efficient, with lower resistance and uniform temperature distribution. Leveraging biomimetic design principles further improves the reliability and energy efficiency of the cooling system, ensuring robust performance under various complex operating conditions.

##### Cold Plates Inspired by the Limulus Structure

As illustrated in Figure 16(f), the shapes of marine organisms that float in water are closely related to minimizing water flow resistance, a result of millions of years of evolution. Drawing inspiration from this, biomimetic horseshoe crab fins proposed a marine biology‐based design, considering various design variables such as fin geometry, positioning, and inlet/outlet arrangements to enhance the heat dissipation performance of the liquid. The cooling plate aims to reduce the pressure loss of the coolant within the plate. Compared to rectangular and elliptical conventional fin shapes, Zhang et al. demonstrated that the biomimetic horseshoe crab fins significantly improved ΔP and average temperature.^[^ ^108^ ^]^ Multi‐objective optimization was conducted for the parameters of the secondary channel. The optimized model showed a reduction of average temperature and ΔP by 1.69 °C (4.61%) and 6.81 Pa (54.26%), respectively.^[^ ^108^ ^]^

##### Cold Plates Inspired by Human Vascular Structures

As shown in Figure16(g), Li et al. designed a biomimetic vascular structure for liquid cooling plates in power battery systems, drawing on biomimetic principles and human vascular models to provide favorable temperatures.^[^ ^109^ ^]^ Under 3C discharge, the T_max_ of the liquid cooling plate reached 31.88 °C, with a temperature difference of 4.10 °C.^[^ ^109^ ^]^

##### Cold Plates Inspired by Natural Nasturtium Textures and Honeycombs

An et al. proposed a hybrid BTMS featuring dual biomimetic cooling plates inspired by natural nasturtium textures and honeycombs.^[^ ^110^ ^]^ By combining biomimetic cooling channels with honeycomb plates, the system enhanced heat dissipation and mass grouping. Under a 3C discharge rate and 40 °C temperature, the T_max_ of the battery decreased by 1.46%, the temperature difference reduced by 13.92%, and the pressure differential across the flow channels decreased by 40.28%.^[^ ^110^ ^]^ In harsh environments, the mass grouping rate improved by 9.89%, allowing the optimized hybrid BTMS to achieve peak temperatures of 44.7 °C, a temperature difference of 3.67 °C, a pressure reduction of 84.3 Pa, and a grouping rate of 80.85%.^[^ ^110^ ^]^

##### Cold Plates Optimized by Fish Scale Structures

As shown in Figure16(h), Mu et al. introduced a design method for optimizing the thermal performance of cooling plates using a biomimetic fish scale (BFS) channel structure.^[^ ^111^ ^]^ The study examined the effects of different structural parameters on the cooling performance of the BTMS liquid cooling plate. Under optimal structural parameters, the T_max_ decreased by 1.61 °C (10.8%), and the maximum temperature difference dropped by 0.43 °C (16.7%). Additionally, the outlet flow rate increased by 2.72%, and pressure decreased by 4.98%.^[^ ^111^ ^]^ Consequently, the proposed BTMS demonstrates effective cooling performance under high power consumption. This study presents a novel BTMS with a BFS structure, aiming to provide solutions for optimizing the thermal performance of serpentine channel battery packs in industrial manufacturing.

##### Cold Plates Inspired by Pumpkin Structures

Huang et al. proposed a new method of introducing streamlined concepts into multi‐microchannel cooling plates, with channel shapes resembling pumpkins to enhance the overall cooling performance of lithium‐ion battery thermal management systems.^[^ ^112^ ^]^ The heat exchanger efficiency could be improved by up to 44.52%. The use of streamlined channel models effectively regulates flow smoothness, preventing turbulence and shock flows. Furthermore, the adoption of streamlined channel plates reduces overall flow resistance.

Currently, biomimetic designs in liquid cooling systems primarily focus on the structural design of liquid cooling plates and cooling pipelines within indirect liquid cooling systems, aiming to optimize fluid dynamics and heat transfer performance. Common sources of inspiration include natural structures such as spider webs, plant leaf veins, serpentine forms, and fish scales. These biomimetic designs address the challenges of low heat transfer efficiency, uneven temperature distribution, high flow resistance, and the complexity and cost of traditional liquid cooling systems. For instance, biomimetic spider webs and leaf vein structures improve the heat exchange performance of cooling plates, reducing the adverse effects of air gaps on heat transfer, while biomimetic serpentine designs optimize fluid flow paths, reduce pressure drop, and enhance temperature uniformity. Simultaneously, streamlined biomimetic structures effectively improve heat dissipation efficiency while maintaining efficient cooling at lower power consumption. Through these designs, the overall performance of liquid cooling systems has been significantly enhanced.

#### 3.1.4 Hybrid Cooling Systems

To overcome the limitations of single cooling methods, hybrid systems have been explored, with PCM–active cooling combinations being the most common. This strategy offsets the low thermal conductivity of PCMs, such as paraffin, by combining their passive thermal buffering with the efficient heat dissipation of liquid cooling, enabling stable control under high discharge conditions. However, it also increases system weight and complexity. In cylindrical batteries, poor fin–cell contact often requires additional fins, reducing energy density and complicating assembly. Biomimetic design offers a promising pathway to address these challenges by enhancing heat transfer efficiency without excessive structural or energy costs.

##### Radiator Inspired by Shark Skin Microstructure

**Figure 17:**
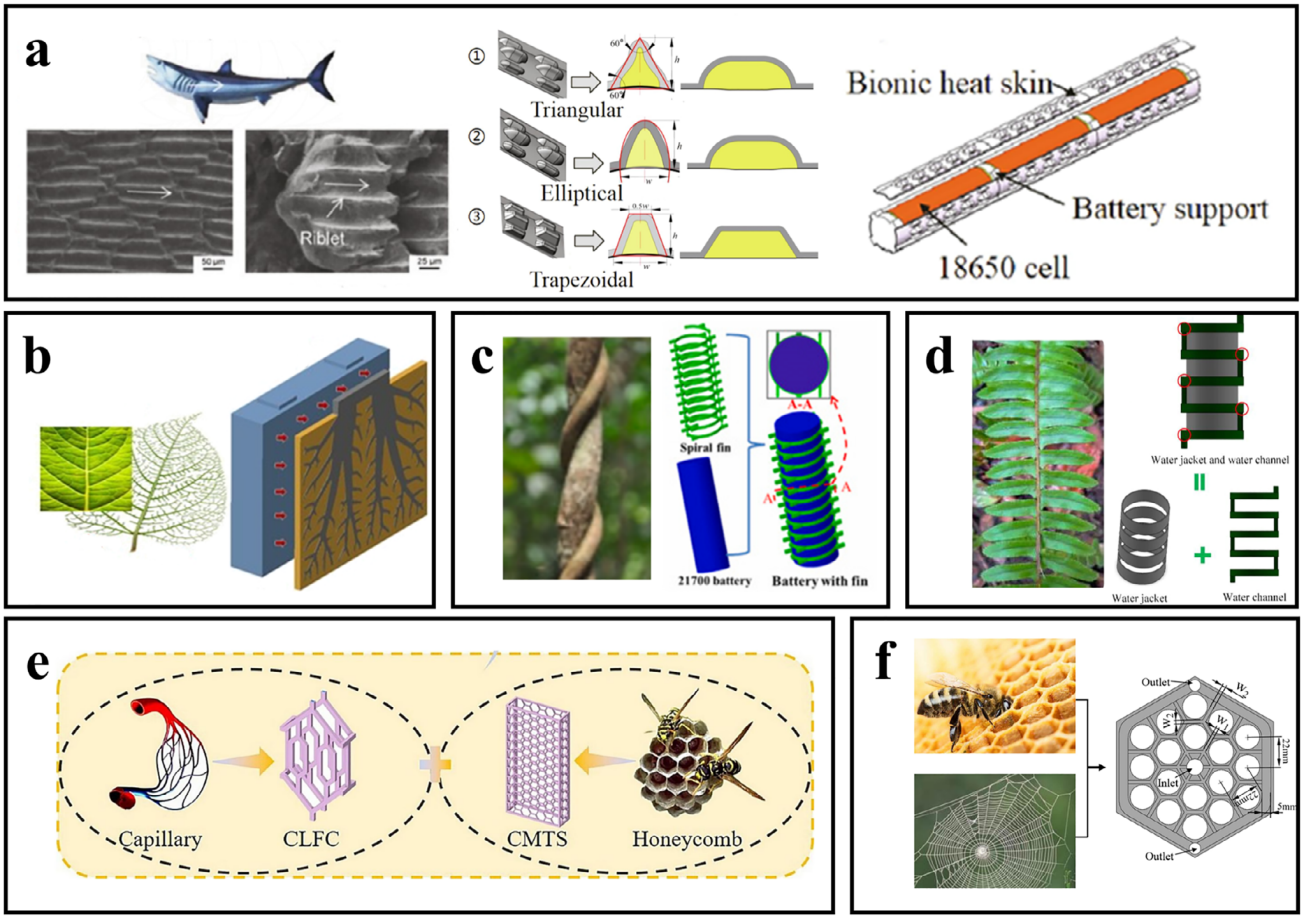
Application and optimization of bio‐inspired design in hybrid cooling BTMS. (a) A heat sink inspired by the microstructure of shark skin. Adaptedwith permission.^[^ ^113^ ^]^ Copyright 2021, Elsevier Ltd. (b) Fin structure design based on leaf venation.  Adapted with permission.^[^ ^94^ ^]^ Copyright 2022, Elsevier Ltd. (c) Bionic spiral fins inspired by vines and spirulina. Adapted with permission.^[^ ^93^ ^]^ Copyright 2024, Elsevier Ltd. (d) Bionic design modeled after the growth patterns of nephrolepis.   Adapted with permission.^[^ ^114^ ^]^ Copyright 2022, Elsevier Ltd. (e) A hybrid battery thermal management system inspired by capillaries and honeycombs.  Adapted with permission.^[^ ^92^ ^]^ Copyright 2024, Elsevier Ltd. (f) A hexagonal cooling plate with integrated bionic liquid microchannels and phase change materials, featuring a honeycomb structure. Adapted with permission.^[^ ^115^ ^]^ Copyright 2021, Elsevier Ltd.

As show in **Figure** **17**(a), inspired by the microstructure of shark skin, Yang et al. proposed a novel heat sink for BTMS, which integrates axial air cooling with PCM.^[^ ^113^ ^]^ The surface of the shark skin‐inspired heat sink features numerous regularly arranged hollow protrusions filled with PCM. The T_max_ and ΔT of the battery module are maintained at 308.98 and 3.21 K, with an energy consumption of 34.57 J. Compared to normal conditions, the T_max_ and ΔT of the shark skin‐inspired water tank are reduced by 5.49 and 4.11 K, respectively.^[^ ^113^ ^]^ Additionally, a method for optimizing the distribution of the biomimetic structure's height was proposed. After seven iterations of optimization, the ΔT and energy consumption of the battery module's biomimetic structure were reduced to 1.22 K and 26.81 J, respectively, while the T_max_ reached 309.19 K. Furthermore, the BTMS demonstrated reliable cooling performance under various environmental temperatures and conditions.^[^ ^113^ ^]^

##### Radiator Inspired by Leaf Vein Structure

As show in Figure 17(b), Liu et al. addressed the low thermal conductivity that limits the application of PCM as a passive thermal management system in battery cooling systems by drawing inspiration from leaf vein structures.^[^ ^94^ ^]^ Leaf veins exhibit a classic branching structure that efficiently absorbs heat in the plant's energy transfer system. To enhance overall thermal conductivity, a PCM battery thermal management system combined with leaf vein fins was proposed. This cooling system consists of biomimetic leaf vein fins and PCM, significantly reducing the melting time of the PCM and achieving better thermal efficiency than traditional parallel designs. The fin holes and surrounding blanks are filled with PCM, which absorbs substantial heat. The leaf vein fin heat sink is connected to the upper cooling channel of the battery pack, allowing for heat exchange between the PCM and the battery through the heat sink with the coolant or environment. The combination of leaf vein fins with PCM effectively dissipates heat from the battery surface and maintains a uniform surface temperature. Comparisons between the battery temperature and PCM melting revealed that the temperature drop of the leaf vein fin cooling system is 34.6% greater than that of traditional rectangular fins.

##### Radiator Inspired by Vines and Spirulina

As show in Figure 17(c), Chen drew inspiration from vines and spirulina to enhance mass exchange efficiency by increasing the contact area, proposing a biomimetic spiral fin.^[^ ^93^ ^]^ This system includes biomimetic spiral fins containing PCM and embedded liquid cooling. Cylindrical batteries are cleverly positioned atop the spiral fins, allowing heat transfer from the battery and PCM through extended small wings to the cold plate. The battery is located within the spiral fins, and strategically placed extended fins along the periphery of the spiral fins facilitate efficient heat conduction to the liquid cooling plate. This configuration serves a dual purpose: controlling temperature within the BTMS and promoting latent heat recovery from the PCM. Compared to the BTMS without fins, T_max_ significantly decreases by 2.4 °C. Additionally, when the liquid cooling operates for 600 s at a flow rate of 0.09 g s^−1^, the liquid fraction of PCM drops from 57% to 26%, maintaining T_max_ at 38.1 °C, which represents a decrease of 3.1 °C compared to the BTMS without fins. Furthermore, when the flow directions of the cold plates are opposite, T_max_ is reduced by 0.8 °C.^[^ ^93^ ^]^

##### Radiator Inspired by the Growth Pattern of Nephrolepis

As show in Figure 17(d), An et al. took inspiration from the growth pattern of nephrolepis, where the stems and leaves exhibit axial symmetry that facilitates water flow within the plant body.^[^ ^114^ ^]^ Water flows through a broader area of the plant, allowing for better absorption from the roots. Under conditions of an ambient temperature of 303.15 K and a mass fraction of ≈40% for the composite PCM, the adjustable maximum temperatures are found to be 310.28 and 309.99 K, with inter‐battery temperature differences of 4.06 and 4.21 K for discharge rates of 4C and 5C, respectively. Nephrolepis can withstand both high and low temperatures, surviving even at 238.15 K.^[^ ^114^ ^]^

##### Battery Thermal Management System Inspired by Capillary and Honeycomb Structures

As show in Figure 17(e), An et al. also proposed an innovative capillary/honeycomb hybrid battery thermal management system (CH‐HBTMS) that combines biomimetic cooling channels with honeycomb cold plates to improve the heat dissipation and mass grouping efficiency of square LIBs.^[^ ^92^ ^]^ The multi‐branch capillary structure coupled with honeycomb enhances the contact area and distribution uniformity between the flow channels and the battery, thus improving heat dissipation capabilities and facilitating rapid heat transfer from the battery.Compared to traditional liquid cooling plates, the CH‐HBTMS achieves a 14.11% improvement in mass grouping rate. The optimized CH‐HBTMS ensures that under harsh conditions, the battery temperature rise is only 3.38 °C, with the temperature difference between the cells remaining below 2.84 °C, demonstrating excellent heat dissipation performance.^[^ ^92^ ^]^

##### Hexagonal Cooling Plates Inspired by Spider Webs and Honeycomb Structures

As shown in Figure 17(f), Yang et al. proposed a novel honeycomb BTMS that integrates biomimetic liquid microchannels with phase change materials in a hexagonal cooling plate.^[^ ^115^ ^]^ The biomimetic liquid microchannels in the cooling plate draw inspiration from spider webs and honeycomb structures. Under conditions of a discharge current of 32.2 A, an inlet flow rate of 0.001 kg s^−1^, and an ambient temperature of 298.15 K, the T_max_ of the battery module using an internal flow configuration reached 309.15 K, with a ΔT of 3.8 K and a pressure drop of 11.95 Pa. The proposed battery module effectively avoids temperature control failure when the PCM fully melts.^[^ ^115^ ^]^

The hexagonal heat sink incorporates biomimetic liquid microchannels inspired by spider webs and honeycomb structures. The coolant flows into the battery module from the inlet, absorbing the heat generated by the battery and subsequently returning to the reservoir. When the heat absorption rate of the coolant is lower than the heat generation rate of the battery, the PCM absorbs the excess heat.

Overall, these biomimetic designs significantly increase the contact area between the battery and the cooling medium, enhancing thermal conductivity and reducing the failure of phase change materials. Furthermore, these designs replicate natural heat dissipation mechanisms, leading to improved temperature control, decreased internal temperature differences within the battery, and reduced energy consumption. This approach notably enhances the stability and efficiency of the system.

### 3.2 Fire Protection Designs

The external protective layer of power batteries enhances safety, structural strength, and environmental adaptability across three levels: cell, module, and pack. At the cell level, metal or plastic enclosures provide sealing, moisture and dust protection, mechanical strength, and sometimes heat dissipation. Module protection layers, often aluminum alloy or steel, secure cells against vibration, integrate with cooling systems, and ensure electrical insulation. Pack‐level enclosures, typically lightweight high‐strength alloys or composites, deliver comprehensive mechanical, thermal, and environmental protection, including impact absorption, waterproofing, fire resistance, and corrosion resistance. Closely integrated with cooling technologies, they maintain temperature stability, prolong service life, and ensure overall system safety.

#### 3.2.1 Battery Protection Layers

Battery failures are often related to mechanical‐thermal coupling behavior, necessitating protective shielding materials with excellent mechanical robustness and flame retardancy to mitigate TR. However, most thermal insulation materials lack the strength needed to protect batteries from mechanical abuse, which is one of the most severe situations and can lead to catastrophic consequences. Wood is an emerging candidate material that offers exceptional mechanical and thermal insulation properties due to its layered structure and well‐oriented matrix.

**Figure 18:**
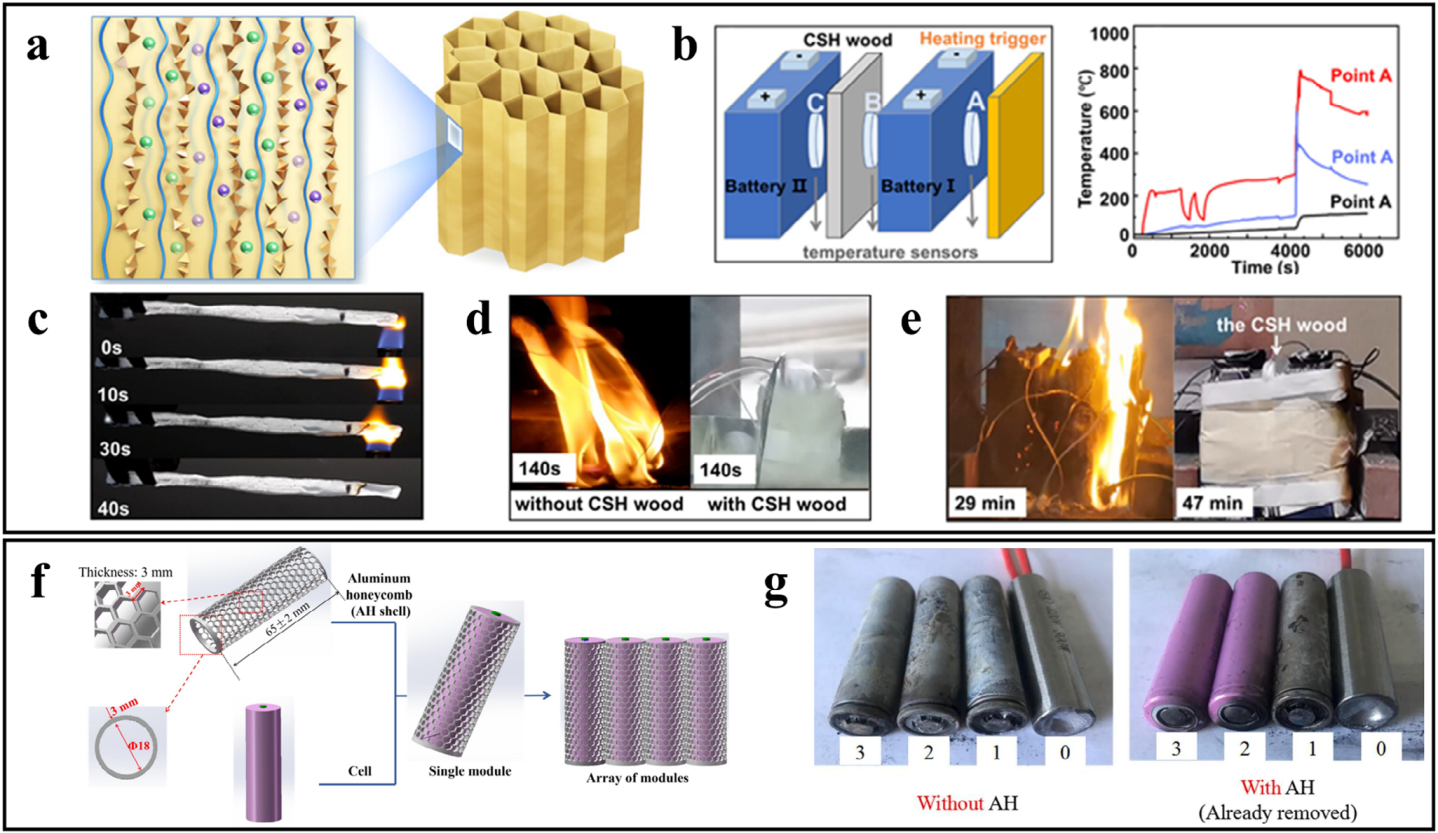
Application of bio‐inspired design in BTMS protective layers. (a) CSH composites modeled after the microstructure of natural wood. Adapted with permission.^[^ ^36^ ^]^ Copyright 2023, Wenzhou University and John Wiley & Sons Australia, Ltd. (b) Temperature sensor placement in the battery pack and temperature variations during the explosion of two lithium battery packs using CSH wood. Reproduced with permission.^[^ ^36^ ^]^ Copyright 2023, Wenzhou University and John Wiley & Sons Australia, Ltd. (c) Horizontal burning test of CSH wood. Reproduced with permission.^[^ ^36^ ^]^ Copyright 2023, Wenzhou University and John Wiley & Sons Australia, Ltd. (d) Temperature change during the explosion of a single lithium battery with CSH wood. Reproduced with permission.^[^ ^36^ ^]^ Copyright 2023, Wenzhou University and John Wiley & Sons Australia, Ltd. (e) Comparison of two battery packs, one with and one without CSH wood. Reproduced with permission.^[^ ^36^ ^]^ Copyright 2023, Wenzhou University and John Wiley & Sons Australia, Ltd. (f) Conceptual design of a cell module wrapped in aluminum honeycomb. Reproduced with permission.^[^ ^116^ ^]^ Copyright 2021, Elsevier Ltd. (g) Battery modules following thermal cycling tests. Reproduced with permission.^[^ ^116^ ^]^ Copyright 2021, Elsevier Ltd.

Inspired by wood, Du et al. developed an effective method for designing layered nanocomposites through the self‐assembly of hydrated calcium silicate and polyvinyl alcohol polymer chains, referred to as calcium silicate hydrate (CSH) wood (as shown in **Figure** **18**(a)).^[^ ^36^ ^]^ Experimental tests on its thermal barrier properties demonstrated that when a heating trigger was applied at end A, the CSH wood effectively reduced the temperature at point C on the surface of battery II (as illustrated in Figure 18(b)). Additionally, to further assess the flame retardancy of CSH wood, a series of combustion tests were conducted, including the horizontal burning test, temperature change analysis of a single lithium battery explosion with CSH wood, and comparisons between battery packages with and without CSH wood (as shown in Figure 18(c–e)).

The multifunctional protective material CSH wood exhibits an unprecedented combination of lightweight properties, high stiffness, negative Poisson's ratio, and outstanding toughness, along with excellent thermal insulation and flame retardancy (UL94‐V0). When used as a protective cover or layer within a battery pack, the robust CSH wood can withstand high impact loads and inhibit thermal diffusion, thereby preventing or delaying the spread of fire and significantly reducing the risk of property damage or personal injury from battery explosions.

#### 3.2.2 Single‐Cell Protective Enclosures

In practical applications, LIBs are typically arranged in compact multi‐cell groups or arrays. If one cell inadvertently experiences TR, this process may propagate to other cells within the group or array. As the propagation speed increases, the situation can become increasingly severe, leading to a higher likelihood of battery fires and explosions.

Therefore, there is an urgent need to develop BTMS with fire‐retardant functions and/or mitigation capabilities for TR and its propagation. Lightweight aluminum honeycomb (AH) structures have shown superior performance in many industrial fields. Weng et al. proposed an AH‐wrapped battery module design, conducting a series of experimental studies on battery modules with and without AH under various conditions and scenarios (as shown in Figure 18(f,g)), which displays TR experiments for battery modules with and without AH).^[^ ^116^ ^]^ The study employed infrared imaging to investigate the impact of AH on thermal management and the propagation of TR, based on theories of heat transfer, including conduction, convection, and radiation, along with TR behavior models of LIBs.

Results showed that the AH structure significantly improved thermal dissipation and TR protection. The wrapped honeycomb module achieved superior cooling under both natural and forced convection, with forced ventilation lowering temperatures by 17.1% compared to the unwrapped module.^[^ ^116^ ^]^ The multi‐hexagonal structure increased surface area, enhancing convective heat transfer, while coupling with PCM further boosted thermal efficiency and reduced battery temperature. Under extreme heating, the wrapped module effectively suppressed TR propagation: although the triggered cell underwent TR, heat did not spread to neighboring cells, and the peak temperature was 63.7 °C lower than in the unwrapped module.^[^ ^116^ ^]^ Overall, the AH structure consistently prevented TR propagation across different charging states.

## 4 Conclusion and Outlook

Thermal safety concerns in power battery systems remain a critical bottleneck constraining the development of the electric vehicle industry. Despite significant advances in both intrinsic and systematic safety strategies, the risk of TR continues to escalate exponentially with increasing battery energy density. In this context, biomimetic design offers innovative approaches for enhancing battery safety. By emulating biological adaptation mechanisms found in nature, researchers have developed diverse biomimetic strategies in material design, structural optimization, and functional regulation, significantly improving battery thermal management efficiency while reducing TR probability.

The first three sections of this study comprehensively review existing thermal safety control technologies and analyze the application of biomimetic design in battery thermal safety from both intrinsic and systematic safety perspectives. Regarding intrinsic safety, research focuses on optimizing internal battery materials to mitigate TR risks. Through biomimetic strategies such as mimicking NK cell anti‐tumor mechanisms, biological porous structures, and self‐healing mechanisms, researchers have successfully enhanced the thermal stability and mechanical strength of battery materials. Specifically, the implementation of high‐temperature composite separators, flame‐retardant functional additives, and protective coating technologies has achieved source‐level suppression of TR.

From a system safety perspective, protection strategies primarily enhance overall battery safety through external design and management mechanisms. Biomimetic strategies play a particularly prominent role in thermal management systems, such as emulating efficient heat dissipation structures found in nature to improve battery cooling efficiency and implementing intelligent response mechanisms for rapid intervention during temperature anomalies. Additionally, biomimetic design contributes significantly to enhancing mechanical protection of battery structures, such as optimizing battery casing impact resistance through biological structure simulation.

**Figure 19:**
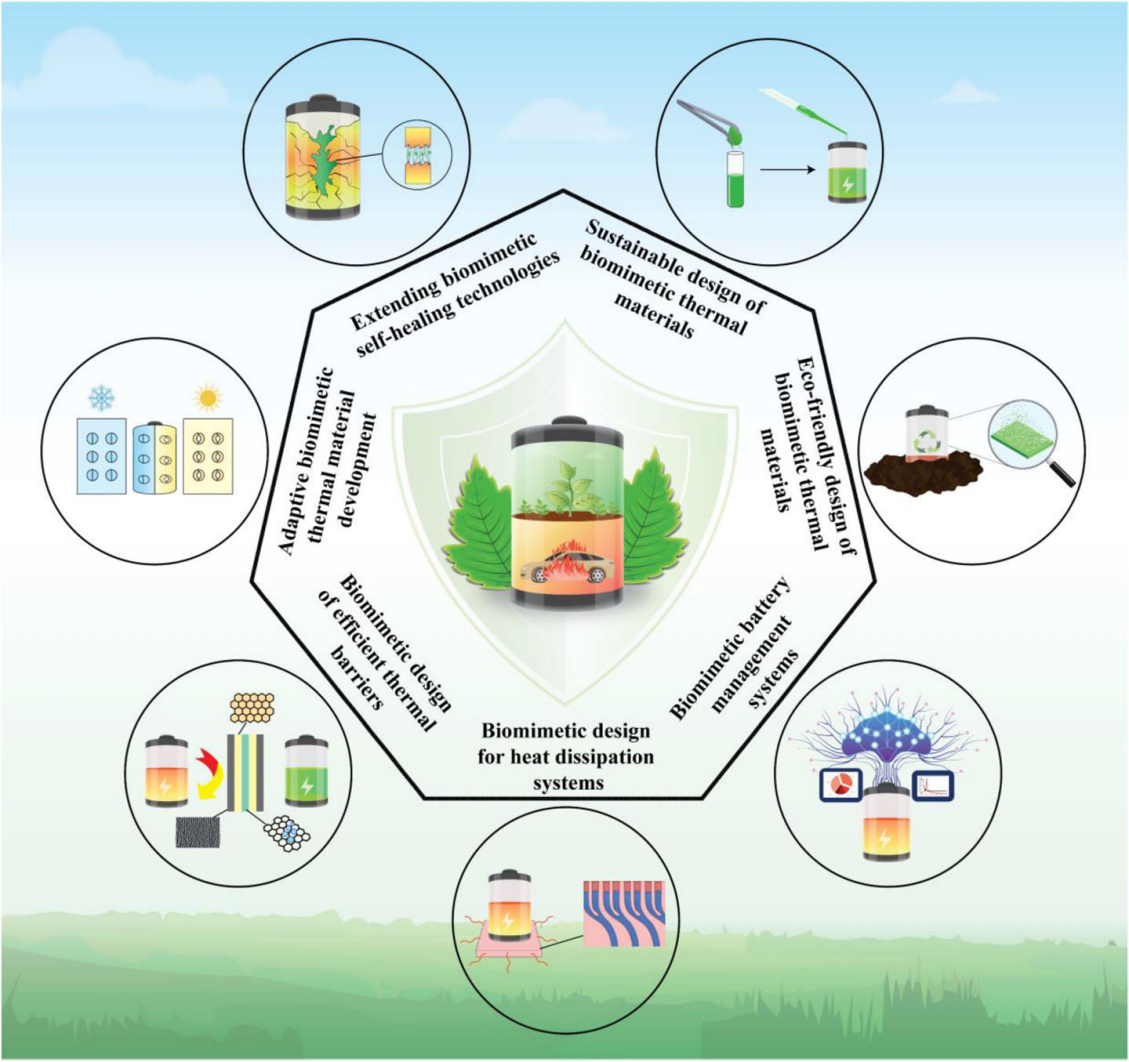
Future biomimetic thermal safety strategies for electric vehicle batteries and engineering approaches.

Despite breakthrough advances in power battery thermal safety through biomimetic design, several technical bottlenecks persist, including how to further enhance material thermal safety while maintaining battery performance and how to achieve synergistic integration of multiple biomimetic strategies. This study outlines several potential future development directions, as illustrated in **Figure** **19**.

### 4.1 Extending Biomimetic Self‐Healing Technologies

Drawing inspiration from biological self‐healing mechanisms (such as human skin), next‐generation power batteries could incorporate rapid self‐healing functions against thermal damage, beyond conventional mechanical degradation repair. Self‐healing electrolytes and separators, designed to autonomously repair localized damage upon early TR indicators, could effectively suppress failure propagation. Such mechanisms significantly reduce TR probability through active damage mitigation at the material level.

### 4.2 Adaptive Biomimetic Thermal Material Development

The integration of biomimetic design with smart materials could lead to more intelligent thermal management systems for future power batteries. For example, adaptive materials can automatically adjust the battery's heat dissipation based on temperature changes, similar to how animals regulate temperature by sweating through pores or how plants open stomata to evaporate water and lower temperature. These materials optimize heat dissipation through phase changes or structural adjustments. At normal temperatures, they maintain a dense structure to minimize heat loss; at higher temperatures, the material surface undergoes morphological changes, such as the formation of porous structures, significantly enhancing heat dissipation. This adaptability effectively controls surface temperature and reduces the risk of local overheating and TR.

### 4.3 Biomimetic Design of Efficient Thermal Barriers

Biomimetic insulation designs can be applied to battery modules to develop more efficient thermal barrier materials. Drawing inspiration from firebreak tree species (e.g., cork oak), bio‐inspired thermal insulation technologies can prevent the spread of heat, thus mitigating TR. Specifically, a bio‐inspired multilayer composite structure can be designed with three layers: an outer layer of non‐combustible material to block flame propagation, a middle porous layer to resist heat diffusion, and an inner layer of modified materials to capture free radicals and prevent further flame ignition. This strategy significantly reduces the risk of heat transfer to adjacent battery cells, enhancing the overall safety of the system.

### 4.4 Biomimetic Design for Heat Dissipation Systems

Natural thermal management systems—exemplified by plant vascular networks and animal circulatory systems—provide crucial insights for optimizing battery cooling mechanisms. Biomimetic design principles applied to liquid cooling systems and thermal channel architectures significantly enhance heat dissipation efficiency while reducing energy consumption and promoting uniform temperature distribution across battery modules. Such biologically inspired optimization effectively mitigates localized overheating and thermal heterogeneity‐induced risks, thereby enhancing system stability and reliability.

### 4.5 Biomimetic Battery Management Systems

Future battery management systems could emulate biological neural networks to develop intelligent control systems with self‐learning and adaptive capabilities. By integrating advanced multi‐functional sensors—such as fiber‐optic temperature probes, gas sensors, acoustic emission detectors, and nano‐enabled pressure sensors—the system can achieve high‐fidelity, real‐time perception of electrochemical and thermal states. These sensory inputs act as the “neural receptors” of the biomimetic network, enabling precise risk assessment and rapid early‐stage intervention against TR events. Analogous to biological neural mechanisms, the system achieves predictive control through multi‐dimensional data analysis before TR initiation. This biomimetic intelligent management strategy offers novel approaches for battery safety and stability, advancing battery technology toward enhanced efficiency and reliability.

### 4.6 Eco‐Friendly Design of Biomimetic Thermal Materials

As environmental regulations become increasingly stringent, the design of future power batteries must not only enhance performance but also consider the recyclability and environmental compatibility of the materials. Biomimicry offers significant insights for the development of biodegradable battery materials. For instance, certain bio‐inspired thermal materials can naturally degrade under specific conditions, thus substantially reducing the ecological pollution caused by discarded batteries. This design approach effectively addresses the growing environmental concerns and lays a foundation for the sustainable development of battery technology.

### 4.7 Sustainable Design of Biomimetic Thermal Materials

The finite nature of global energy and resources drives the development of bio‐inspired thermal materials. By extracting renewable materials from specific plant or biological structures, green approaches for thermal technologies are possible. Research is still in the early stages, and large‐scale application requires further exploration. Recycling and reuse of these materials also offer a sustainable path, extending material lifespan and reducing resource consumption and environmental impact, thereby supporting the green development of power battery technologies.

## Conflict of Interest

The authors declare no conflict of interest.
